# A symptom-based approach in predicting ECT outcome in depressed patients employing MADRS single items

**DOI:** 10.1007/s00406-021-01301-8

**Published:** 2021-07-16

**Authors:** Luisa Carstens, Corinna Hartling, Anna Stippl, Ann-Kathrin Domke, Ana Lucia Herrera-Mendelez, Sabine Aust, Matti Gärtner, Malek Bajbouj, Simone Grimm

**Affiliations:** 1 Department of Psychiatry, Centre for Affective Neuroscience (CAN), Corporate Member of Freie Universität Berlin, Humboldt-Universität zu Berlin, Berlin Institute of Health, Charité-Universitätsmedizin Berlin, Campus Benjamin, Franklin, Hindenburgdamm 30, 12203 Berlin, Germany; 2 Department of Psychiatry, Psychotherapy and Psychosomatics, Psychiatric Hospital, University of Zurich, Lenggstrasse 31, 8032 Zurich, Switzerland; 3 Department of Psychology, MSB Medical School Berlin, Rüdesheimer Str. 50, 14197 Berlin, Germany

**Keywords:** Electroconvulsive therapy (ECT), Montgomery–Åsberg Depression Rating Scale (MADRS), Depression, Response prediction

## Abstract

Establishing symptom-based predictors of electroconvulsive therapy (ECT) outcome seems promising, however, findings concerning the predictive value of distinct depressive symptoms or subtypes are limited; previous factor-analytic approaches based on the Montgomery–Åsberg Depression Rating Scale (MADRS) remained inconclusive, as proposed factors varied across samples. In this naturalistic study, we refrained from these previous factor-analytic approaches and examined the predictive value of MADRS single items and their change during the course of ECT concerning ECT outcome. We used logistic and linear regression models to analyze MADRS data routinely assessed at three time points in 96 depressed psychiatric inpatients over the course of ECT. Mean age was 53 years (SD 14.79), gender ratio was 58:38 (F:M), baseline MADRS score was *M* = 30.20 (SD 5.42). MADRS single items were strong predictors of ECT response, remission and overall symptom reduction, especially items 1 (apparent sadness), 2 (reported sadness) and 8 (inability to feel), assessing affective symptoms. Strongest effects were found for regression models including item 2 (reported sadness) with up to 80% correct prediction of ECT outcome. ROC analyses were performed to estimate the optimal cut-point for treatment response. MADRS single items during the course of ECT might pose simple, reliable, time- and cost-effective predictors of ECT outcome. More severe affective symptoms of depression at baseline and a stronger reduction of these affective symptoms during the course of ECT seem to be positively associated with ECT outcome. Precise cut-off values for clinical use were proposed. Generally, these findings underline the benefits of a symptom-based approach in depression research and treatment in addition to depression sum-scores and generalized diagnoses.

**Supplementary Information:**

The online version contains supplementary material available at 10.1007/s00406-021-01301-8.

## Introduction

Electroconvulsive therapy (ECT) is one of the most effective treatment options for depressive disorders, recommended especially for the treatment of severe and treatment-resistant depression [1, 2]. Even though response rates are generally high (60–80%) [3], a relevant percentage of patients shows no or only partial response [4]. Moreover, response time and course of action during ECT vary substantially [4]. Different mechanisms of actions are discussed in the literature (e.g. neurobiological factors such as enhancement of serotonergic neurotransmission and activation of the mesocorticolimbic dopamine system) [3]. However, the precise antidepressant mechanisms of ECT remain unclear, potentially further impeding treatment prediction [5]. Generally, electroconvulsive therapy can be regarded as a relatively costly, intensive treatment, implying patients being hospitalized for several weeks undergoing recurrent anaesthesia. Moreover, transient cognitive side effects and the psychological distress for patients undergoing this treatment without the desired effects need to be considered [6, 7]. At the same time, there is well-established evidence that ECT is a very effective treatment option for severely depressed patients with a long history of treatment efforts [1]. These factors underline the importance of establishing reliable response predictors of antidepressant treatment with ECT. Factors such as age, psychotic symptoms and depression severity appear to be well-founded predictors of successful ECT treatment; however, findings concerning depression symptoms or subtypes are less clear [8]. In their factor-analytic approaches, Okazaki et al. [9] Tominaga et al. [10] and Spashett et al. [11] examined factors derived from the Montgomery–Åsberg Depression Rating Scale (MADRS) [12] as predictors of ECT response. Postulating response predictors employing one of the most established clinical interviews for depression severity seems of great value for clinical decision making, however, proposed factor models varied across samples and implications remained inconclusive. Hence, the current study refrains from this factor-analytic approach and rather aims to examine the value of MADRS single items as predictors of ECT response. As this is the first study examining MADRS single items during the course of ECT, we not only aspire to examine the predictive value of MADRS single items and, therefore, depressive symptoms but also seek to depict the change of these depressive symptoms during the course of ECT to deepen the understanding of the antidepressant mechanisms of ECT.

## Method

### Participants

Participants were psychiatric inpatients diagnosed with a current depressive episode in accordance with DSM-5 who were treated with ECT at Charité—Universitätsmedizin Berlin. The present study analyses routinely assessed depression severity employing the MADRS [12], so no restrictions concerning comorbidities or medication intake were made and no clinical trials registration is available. However, the retrospective study design was approved by the institutional review board of the Charité, performed in accordance with the Declaration of Helsinki and patients’ informed consent was obtained. Routine MADRS ratings were available for 120 moderately to severely depressed inpatients, additional clinical and demographic data was collected from medical records. To facilitate interpretation and enhance comparability with other studies, patients who received ketamine treatment right before ECT, changed to ketamine treatment during the course of ECT or received ketamine as an anaesthetic during ECT were excluded (*n* = 10), as well as patients older than 80 years (*n* = 4), patients who had to pause ECT due to urgent other medical reasons (*n* = 2), were rehospitalized shortly after release and received a second course of ECT (*n* = 1), received only one ECT session per week in the beginning (*n* = 1) or received very few (6 or 7) ECT sessions (*n* = 2). Patients with baseline MADRS total score 2 SD > Mean were identified as outliers and excluded (*n* = 4), resulting in our total sample size of *n* = 96.

### ECT treatment

ECT was administered in accordance with standard protocol at the Department of Psychiatry, Charité—Universitätsmedizin Berlin, which includes three ECT sessions per week (for details see Basso et al. [13] and Brakemeier et al. [14]). In short, patients were anesthetized either with etomidate (approximately 0.75 mg/kg) or propofol (approximately 1.5 mg/kg). A Thymatron IV System (Somatics, LLC, Venice, Florida, United States) was used to deliver ultra-brief pulse stimuli (0.3 ms) for right unilateral ECT. Succinylcholine (approximately 0.75 mg/kg) was used for muscular relaxation. Motor and electroencephalogram (EEG) seizure duration, ictal-EEG wave amplitude and post-ictal suppression index were monitored for seizure quality. During the first ECT session, seizure threshold was titrated and voltage was subsequently modified if patients showed insufficient seizures. The mean number of administered ECT sessions was 13.60 (SD 2.66).

### Study design and assessment

A routinely assessed German version of the Montgomery–Åsberg Depression Rating Scale (MADRS) [12] conducted by trained professionals at baseline before ECT treatment (T0), mid-treatment after six ECT sessions (T1) and at the end of treatment 1–3 days after the last ECT session (T2) was analysed. The MADRS consists of ten items assessing the following depressive symptoms on a seven-point scale: apparent sadness, reported sadness, inner tension, reduced sleep, reduced appetite, concentration difficulties, lassitude, inability to feel, pessimistic thoughts and suicidal thoughts. Reduction of MADRS total score of 50% or more at T2 was defined as response, MADRS total score ≤ 10 at T2 as remission, 50% reduction or more at T1 was defined as early response [15]. In our sample, 53% of the patients responded, 34% remitted, all patients who remitted responded as well, 24% were classified as early responders.

### Statistical analyses

All analyses were conducted using SPSS^®^ 26.0 (IBM Corporation, Armonk NY, USA) for Windows^®^/Apple Mac^®^. *T* tests for independent samples were used to examine differences between responders and non-responders concerning clinical or demographic variables, chi-squared tests were used to assess differences between categorical variables. As distribution of gender differs between responders and non-responders, gender was added to the regression models.

#### Change of depressive symptoms during the course of ECT

ANOVAs for repeated measures (T0, T1, T2) were applied, separately for all single items and MADRS total score, gender and psychotic symptoms were added as covariates. These ANOVAs were performed for the overall sample and separately for responders and non-responders. For the overall sample, classification as responders vs. non-responders was additionally added as a covariate.

#### Prediction of response

A two-step logistic regression model was used to predict response. In order to control for gender, psychotic symptoms and number of received ECT sessions these three variables were added in the first step, in the second step, MADRS single items and MADRS total score were each added to a distinct model individually, thus each regression model consisted of gender, psychotic symptoms, number of ECT sessions in the first step and one MADRS item (or MADRS total score) in the second step. In the second step interaction terms of the respective MADRS item or total score with gender, psychotic symptoms and number of ECT sessions were added as well, these were removed when not significant. In addition to the predictive value of MADRS items and total score at baseline (T0) and mid-treatment (T1), we also examined the predictive value of the change scores. Change scores T0:T1 are defined as the change in percentage from T0 to T1, change scores T0:T2 as the change in percentage from T0:T2.

#### Prediction of early response

The same two-step logistic regression model as described above was applied.

#### Prediction of remission

Considering the relatively small amount of remitted patients (34%) in our sample, the fact that in the group of 51 responders all 31 remitters are included, and that from a clinical perspective we consider response prediction to be a more urgent matter, we decided to only briefly report remission prediction here, the same two-step logistic regression model as described above was applied.

#### Prediction of overall symptom reduction

Even though the response definition of 50% symptom reduction is well established, this dichotomisation can be regarded as a rough simplification which undoubtedly implies loss of information. Thus, we decided that an important criterion for successful ECT treatment is not only response, but also overall symptom reduction, which we defined as change of MADRS total score in percentage from T0 to T2 (change score MADRS total score T0:T2). To predict overall symptom reduction, we used a two-step linear regression model, similar to the logistic regression model mentioned above. To control for gender, psychotic symptoms and number of ECT sessions these three variables were added in the first step, in the second step, MADRS single items and MADRS total score were each added to a distinct model individually.

#### ROC curves

Additionally, receiver operating characteristic (ROC) analyses were performed to estimate the optimal cut point for MADRS items and total score at baseline for response at the end of treatment.

All *p* values are Bonferroni-corrected where applicable, except for T0 as predictor of ECT response. All assumptions of the respective tests were satisfied or it was reasonable to conclude that the tests were robust against the respective violations, thus only parametric tests were used. Normality of distribution was tested with the Shapiro–Wilk test, equality of error variances was tested with Levene’s test, Greenhouse–Geisser correction was applied were necessary. Cohen’s *f*, Cohen’s *d,* Phi coefficient (*φ*)*,* or partial *η*^2^ are reported as effect sizes.

## Results

### Clinical and demographic data

Our sample consisted of *n* = 96 psychiatric inpatients diagnosed with a depressive episode. Demographic and clinical characteristics for the overall sample, responders and non-responders are shown in Table 1. In our overall sample, 47% of the inpatients were diagnosed with psychiatric comorbidities, 67% received concomitant antidepressant medication. For detailed description of diagnosis type, psychiatric comorbidities and antidepressant medication, please see Tables 2 and 3, supplementary material.

**Table 1:** Demographic and clinical characteristics

Variable	Overall sample			Responders			Non-responders			*t*	*df*	*p*	*d*
	*M*	SD	*n*	*M*	SD	*n*	*M*	SD	*n*				
Age	52.60	14.79	96	54.67	15.15	51	50.27	14.18	45	− 1.46	94	0.147	− 0.30
Education (years)	14.05	2.85	88	13.84	2.83	44	14.29	2.93	42	0.72	84	0.476	0.16
Number of psychiatric hospitalizations^a^	3.98	3.33	94	4.18	4.15	50	3.75	2.02	44	− 0.65	73	0.518	− 0.15
Number of depressive episodes	7.24	9.59	46	9.04	11.36	28	4.44	4.97	18	− 1.61	44	0.114	− 0.49
Duration of current episode (months)^a^	9.61	9.44	46	8.53	9.44	27	11.13	9.66	19	0.92	44	0.364	0.28
Baseline (T0) MADRS total score	30.20	5.42	96	31.47	5.34	51	28.76	5.21	45	− 2.52	94	0.014	− 0.52
Mid-treatment (T1) MADRS total score	20.30	7.26	96	16.94	7.02	51	24.11	5.47	45	5.53	94	< 0.001	1.14
Treatment end (T2) MADRS total score	14.30	7.91	96	8.24	4.10	51	21.26	5.01	45	13.88	94	< 0.001	2.86
Change MADRS total score T0:T2	50.91	28.29	96	73.26	13.27	51	25.58	17.12	45	15.34	94	< 0.001	3.16
Number of ECT sessions	13.60	2.66	96	13.27	2.65	51	13.98	2.66	45	1.30	94	0.198	0.27

							*χ*^2^		*p*	*φ*
Gender (F:M)	58:38	96	37:14	51	21:24	45	5.66	1	0.017	0.26
Psychotic Symptoms^b^ (Y:N)	11:85	96	10:41	51	1:44	45	5.51	1	0.019	0.27
Suicide Attempt Lifetime^b^ (Y:N)	30:41	71	17:23	50	13:18	31	0.00	1	0.962	0.01

^a^Assumption of equality of error variances violated (Levene’s test: *p* < 0.05)

^b^Yates corrected. Change MADRS total score T0:T2 is defined as the change in percentage from T0 to T2. *d* = Cohen’s *d,* *φ* = Phi coefficient

### Change of depressive symptoms during the course of ECT

**Fig. 1:**
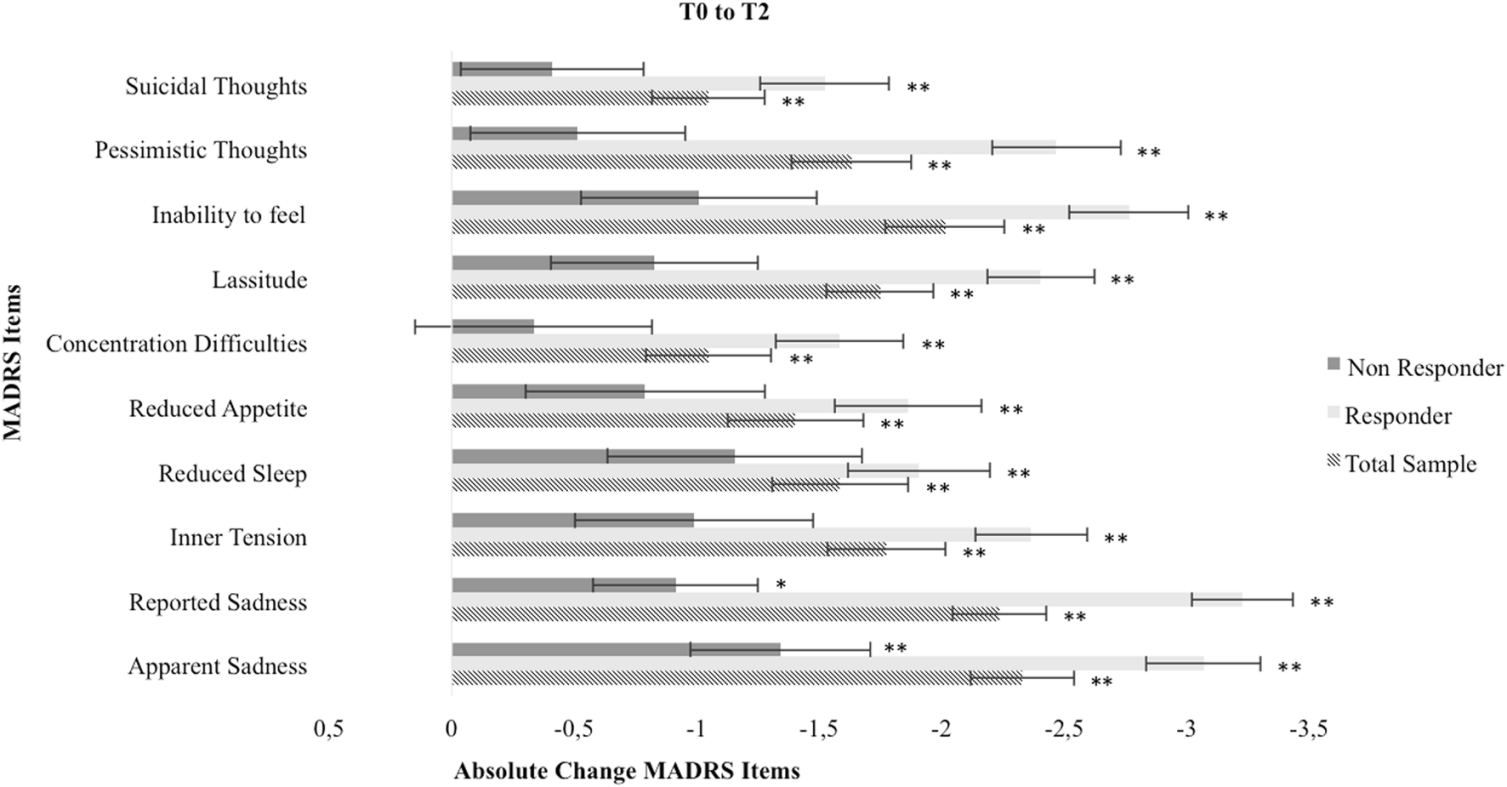
Absolute change of MADRS items from baseline (T0) to treatment end (T2). * = Bonferroni-corrected *p* < 0.05, ** = Bonferroni-corrected *p* < 0.01

**Fig. 2:**
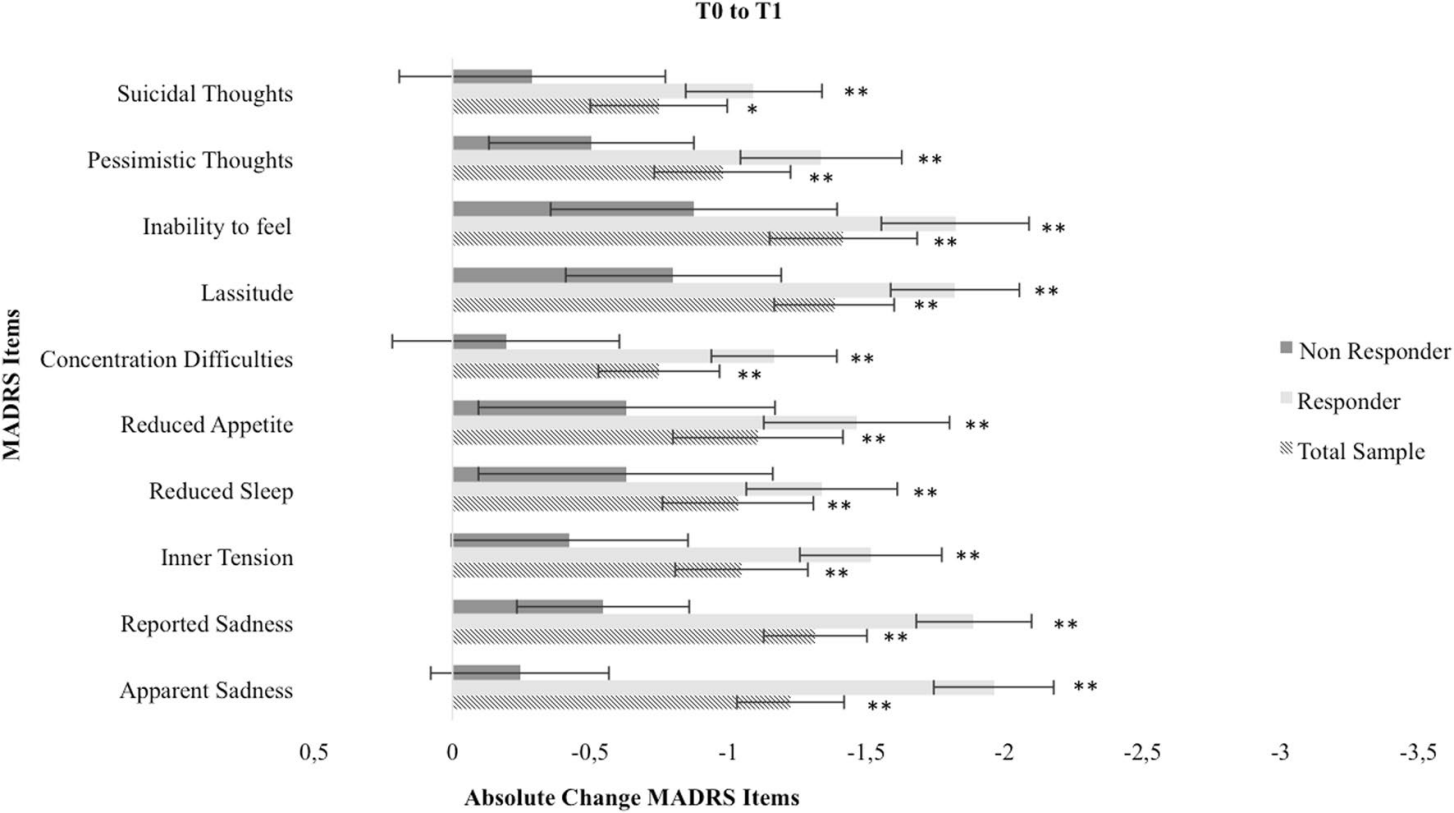
Absolute change of MADRS items from baseline (T0) to mid-treatment (T1). * = Bonferroni-corrected *p* < 0.05, ** = Bonferroni-corrected *p* < 0.01

**Fig. 3:**
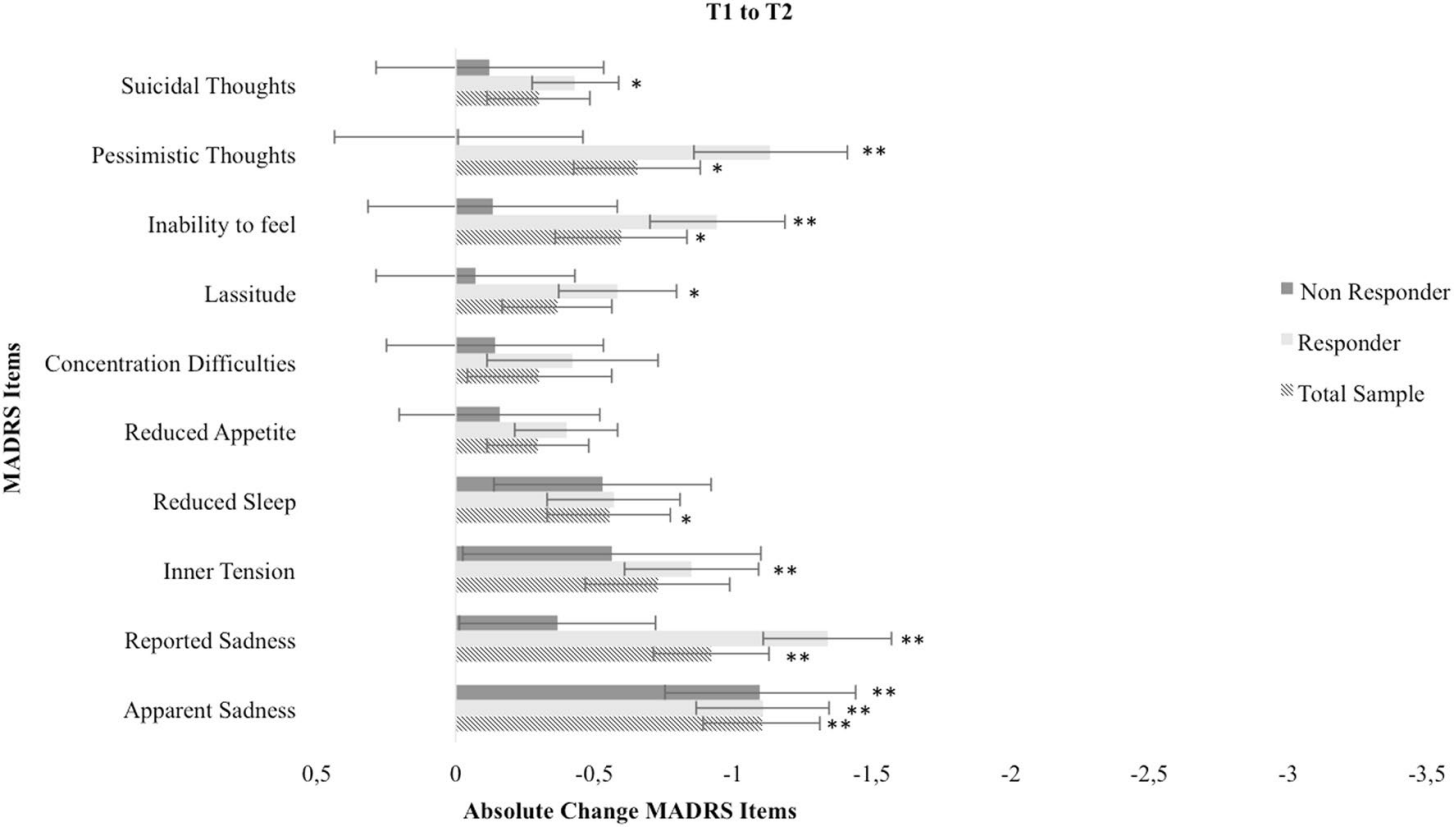
Absolute change of MADRS items from mid-treatment (T1) to treatment end (T2). * = Bonferroni-corrected *p* < 0.05, ** = Bonferroni-corrected *p* < 0.01

ANOVAs for repeated measures (baseline, mid-treatment and treatment end) were performed for the overall sample and within the responder and non-responder group. For the overall sample, all MADRS single items significantly decreased from baseline (T0) to treatment end (T2), Bonferroni-corrected *p* < 0.01. However, decreases within the responder and non-responder group differ, detailed results are shown in Figs. 1, 2 and 3.

### Prediction of response

Stepwise logistic regression showed that in the first step gender (*β* = 1.46, Wald(1) = 9.03, *p* = 0.003, OR 4.32) and psychotic symptoms (*β* = 2.86, Wald(1) = 6.55, *p* = 0.010, OR 17.40) were strongly associated with response, while number of ECT sessions did not contribute significantly to the model (*β* = − 0.13, Wald(1) = 2.37, *p* = 0.124, OR 0.88), *χ*^2^(3) = 20.11, *p* < 0.001, *R*^2^ = 0.25, *f* = 0.58, 68% correct prediction.

In the second step, MADRS single items and MADRS total score were each added to a distinct model individually, thus each model consisted of gender, psychotic symptoms, number of ECT sessions, and one MADRS item (or MADRS total score).

#### Baseline (T0)

MADRS total score (*β* = 0.62, Wald(1) = 4.71, *p* = 0.030, OR 1.88), item 1 (*β* = 0.65, Wald(1) = 4.78, *p* = 0.029, OR 1.91), item 2 (*β* = 4.41, Wald(1) = 6.28, *p* = 0.012, OR 82.10) and item 8 (*β* = 2.59, Wald(1) = 4.67, *p* = 0.031, OR 13.27) were significantly associated with ECT response, the model including item 2 and gender*item 2 interaction showed the best fit: *χ*^2^(5) = 31.60, *p* < 0.001, *R*^2^ = 0.37, *f* = 0.77, 70% correct prediction.

#### Mid-treatment (T1)

MADRS total score (*β* = − 0.17, Wald(1) = 14.48, *p* < 0.001, OR 0.84), item 1 (*β* = − 0.68, Wald(1) = 8.24, *p* = 0.004, OR 0.51) and item 2 (*β* = − 0.67, Wald(1) = 8.83, *p* = 0.003, OR 0.51) were Bonferroni-corrected significant predictors, the model including item 2 showed the best fit: *χ*^2^(4) = 30.64, *p* < 0.001, *R*^2^ = 0.37, *f* = 0.77, 78% correct prediction.

#### Change Scores T0:T1

Change scores for items 1, 2, 8, 10, and MADRS total score were Bonferroni-corrected significant predictors. The two models including item 2 (*β* = − 0.05, Wald(1) = 16.54, *p* < 0.001, OR 0.95), *χ*^2^(4) = 44.08, *p* < 0.001, *R*^2^ = 0.49, *f* = 0.98, 80% correct prediction and MADRS total score (*β* = − 0.07, Wald(1) = 19.03, *p* < 0.001, OR 0.94), χ^2^(4) = 50.56, *p* < 0.001, *R*^2^ = 0.55, *f* = 1.11, 80% correct prediction, showed the best fit.

#### Change Scores T0:T2

Change scores for items 1, 2, 5, 6, 8, 9, and 10 were Bonferroni-corrected significant predictors, the model including item 2 (*β* = − 0.12, Wald(1) = 19.48, *p* < 0.001, OR 0.89) showed the best fit: χ^2^(4) = 89.42, *p* < 0.001, *R*^2^ = 0.81, *f* = 2.06, 92% correct prediction.

Complete information for all regression analyses predicting response can be found in Table 4, supplementary material.

### Prediction of early response

Stepwise logistic regression showed that in the first step psychotic symptoms (*β* = 1.84, Wald(1) = 6.66, *p* = 0.010, OR 6.29) were associated with early response, whereas no effect of gender was found (*β* = 1.06, Wald(1) = 3.32, *p* = 0.069, OR 2.88), *χ*^2^(2) = 9.20, *p* = 0.010, *R*^2^ = 0.14, *f* = 0.40, 49% correct prediction.

In the second step, no effects of MADRS items predicting early response were found (all *p* > 0.05).

Complete information for all regression analyses predicting early response can be found in Table 5, supplementary material.

### Prediction of remission

Stepwise logistic regression showed that in the first step gender (*β* = 1.60, Wald(1) = 8.57, *p* = 0.003, OR 4.97), psychotic symptoms (*β* = 1.44, Wald(1) = 3.96, *p* = 0.047, OR 4.24) and number of ECT sessions (*β* = -0.23, Wald(1) = 5.76, *p* = 0.016, OR 0.79) were strongly associated with remission, *χ*^2^(3) = 17.48, *p* = 0.001, *R*^2^ = 0.23, *f* = 0.55, 69% correct prediction.

In the second step, MADRS single items and MADRS total score were each added to a distinct model individually, thus each model consisted of gender, psychotic symptoms, number of ECT sessions and one MADRS item (or MADRS total score).

Baseline (T0) MADRS total score and MADRS single items could not predict ECT remission. For mid-treatment (T1), Change Scores T0:T1 and Changes Scores T0:T2 as predictors, results were similar to prediction of response, the two models either including MADRS total score or item 2, respectively, showed the best fit. Complete information for all regression analyses predicting remission can be found in Table 6, supplementary material.

### Prediction of overall symptom reduction

Stepwise linear regression showed that in the first step gender (*β* = − 0.25, *t* = − 2.59, *p* = 0.011) and psychotic symptoms (*β* = − 0.27, *t* = − 2.76, *p* = 0.007) significantly contributed to predicting overall symptom reduction, while number of ECT sessions did not significantly contribute to the prediction (*β* = 0.16, *t* = 1.61, *p* = 0.111), *F*(3, 92) = 4.99, *p* = 0.003, *R*^2^ = 0.11, *f* = 0.35.

In the second step, MADRS single items and MADRS total score were each added to a distinct model individually, thus each model consisted of gender, psychotic symptoms, number of ECT sessions and one MADRS item (or MADRS total score).

#### Baseline (T0)

MADRS total score, item 1, 2, 8, and 9 significantly contributed to predicting overall symptom reduction, MADRS total score was a Bonferroni-corrected significant predictor (*β* = − 0.30, *t* = − 3.25, *p* = 0.002), *F*(4, 91) = 6.77, *p* < . 001, *R*^*2*^ = 0.20, *f* = 0.50.

#### Change Scores T0:T1

Change scores for MADRS total score and items 1, 2, 3, 6, 8, and 10 were Bonferroni-corrected significant predictors. The two models including item 2 (*β* = 0.54, *t* = 6.17, *p* < 0.001), *F*(4, 91) = 14.77, *p* < . 001, *R*^*2*^ = 0.37, *f* = 0.77 and MADRS total score (*β* = 0.54, *t* = 6.18, *p* < 0.001), *F*(4, 91) = 14.80, *p* < . 001, *R*^*2*^ = 0.37, *f* = 0.77 showed the best fit.

#### Change Scores T0:T2

Change scores from T0 to T2 for items 2, 4, 5, 7, 8, 9, and 10 were Bonferroni-corrected significant predictors. The model including item 2 (*β* = 1.84, *t* = 6.08, *p* < 0.001) and number of ECT sessions*item 2 interaction (*β* = − 1.04, *t* = − 3.44, *p* = 0.001) showed the best fit: *F*(5, 90) = 55.81, *p* < 0.001, *R*^*2*^ = 0.74, *f* = 1.69.

Complete information for all regression analyses predicting overall symptom reduction can be found in Table 7, supplementary material.

### ROC curves

As regression models including MADRS total score and item 2 showed the best fit, ROC curves were computed for these variables.

#### MADRS total Score baseline (T0)

Area under the curve was 0.64, *p* = 0.017, optimal cut point by Youden-index was MADRS total score = 32 (sensitivity 0.49, specificity 0.73).

#### MADRS item 2 baseline (T0)

Area under the curve was 0.65, *p* = 0.013, optimal cut point by Youden-index was item 2 = 5 (sensitivity 0.47, specificity 0.78).

## Discussion

### Main findings

In this retrospective naturalistic study, we examined 96 psychiatric inpatients diagnosed with a depressive episode who were treated with ECT at Charité—Universitätsmedizin Berlin. We studied change of depressive symptoms during the course of ECT and explored whether depressive symptoms and their change during the course of ECT could predict treatment outcomes. We analysed the routinely assessed MADRS from three time points: baseline (T0), mid-treatment (T1) and end of treatment (T2). For the first time, MADRS single items and their association with ECT outcomes were examined.

For all patients, MADRS total score significantly decreased from baseline to treatment end. Considering the single items, highest reductions were found for items 1 (apparent sadness), 2 (reported sadness) and 8 (inability to feel). Responders showed significant reductions for all single items. Significant reductions for single items within the non-responder group were only found for item 1 (apparent sadness) and 2 (reported sadness).

In our sample, 53% of the patients responded, women were more likely to respond to ECT than men, as were patients who experienced psychotic symptoms during their current episode, age was not associated with response. Responders showed higher depression scores at baseline and lower scores at the end of treatment.

MADRS total score, single items and their change during the course of ECT were useful predictors of ECT response, remission and overall symptom reduction. Single items, especially item 1 (apparent sadness), 2 (reported sadness) and 8 (inability to feel) showed predictive values comparable with or higher than that of MADRS total score. Strongest effects were found for item 2 with large effect sizes, e.g. the regression model including the change of item 2 (reported sadness) from baseline to treatment end showed 92% correct prediction of ECT response or non-response. It is important to note the direction of these effects: at baseline, higher depression scores are positively associated with ECT outcome, at mid-treatment lower depression scores are positively associated with ECT outcome. No effects for prediction of early response were found. ROC curves were computed to estimate the optimal cut point for MADRS total score and item 2 (reported sadness) at baseline for response at the end of treatment: MADRS total score = 32, item 2 = 5.

### Comparison with findings from other studies

In accordance with previous findings, depression severity at baseline and a larger symptom reduction until mid-treatment was positively associated with ECT outcome [8, 16, 17]. Response rate in our sample was relatively low, this might be due to the relatively low percentage of patients with psychotic features and the exclusion of patients diagnosed with schizoaffective disorders in contrast to other studies such as Nordenskjöld et al. [18], as well as differences in electrode placement, dosage and utilized anaesthetic [6]. Even though women were not more severely depressed at baseline and did not report psychotic features more frequently, we found higher response rates for women in our sample, which has not been reported by previous studies [7]. Patients’ age ranged from 22 to 80 years, however, in accordance with some other studies, no association between age and ECT response was found [19, 20]. Contradicting previously reported effects of age were rather small and a possible “turning-point” in the mid-fifties is discussed, however, this remains an open question for further research [8].

Strongest predictive effects were found for item 2 (reported sadness), this can be linked to previous factor-analytic findings. Even though Okazaki et al. [9] and Spashett et al. [11] proposed two distinct predicting factors, consisting of different MADRS single item combinations (called “dysphoria” and “despondency”, respectively), item 2 occurs to be the one item these two distinct factors have in common. ECT outcome was positively associated with a higher affective symptomatology at baseline (item 1 apparent sadness, item 2 reported sadness, item 8 inability to feel) and during the course of ECT these symptoms showed the strongest decrease. Partly, this can be associated with previous findings linking melancholic features of depression to ECT outcome [21]. However, findings remain inconsistent, especially as definitions and assessment of melancholic features vary. Melancholic features often imply a broad variety of differing symptoms, not only affective but also somatic symptoms such as agitation, loss of appetite and sleeping disturbances [7, 8]. We found no evidence for strong predictive values of somatic symptoms for ECT outcome. Our findings underline the proposed limited usefulness of melancholic features as predictor of ECT outcome [22] and support a more symptom-based approach of depression research as proposed by Fried and Nesse [23] corresponding to the heterogeneity of depressive disorders [24].

### Implications

Our findings imply a strong antidepressant effect of ECT, especially in decreasing affective symptomatology. More severely depressed patients seem to benefit more, especially patients reporting pronounced affective symptoms measured with MADRS at baseline. We propose MADRS total score = 32 and item 2 = 5 at baseline as potential cut-off points determined with ROC curves. After future validation and in combination with other aspects such as age, psychotic symptoms, psychomotor symptoms and the physicians’ general assessment, these cut-off points might pose a useful addition to clinical decision-making. MADRS single items and their change during the course of ECT (especially item 2 reported sadness) can provide a simple, reliable, cost- and time-effective contribution to predicting ECT outcome. While patients classified as non-responders also show significant decrease in affective symptoms, responders and non-responders differ particularly concerning the reduction of pessimistic thoughts. Thus, patients who did not sufficiently benefit from ECT might particularly benefit from additional interventions such as cognitive behavioural therapy after ECT. Unfortunately, the limited research examining cognitive behavioural interventions after ECT, while promising, until now mainly focused on maintaining ECT response, disregarding those other patients in need [14, 25, 26].

### Limitations

No follow-up data were available, thus no assumptions about long-term predictive values of MADRS single items regarding maintained response or potential relapse can be made. Due to the naturalistic setting, potential confounding of depressive symptoms with psychiatric medication or comorbidities cannot be ruled out. Future studies with a constant concomitant psychotropic medication might also be enlightening to determine specific effects of ECT. Considering the heterogeneity of depression scales [27], analysing predictive values of depressive symptoms assessed with a different depression scale such as the Hamilton Depression Rating Scale (HRSD) [28] or self-report measures such as the BDI-II (Beck Depression Inventory) [29] seems advisable for robust conclusions. Taking into account the importance of other demographic and clinical predictors such as age and psychomotor symptoms [30], future studies with larger samples might examine more comprehensive regression models including all these factors. This might help to gain a better understanding of their respective, potentially interacting, effects [31].

## Conclusions

In this naturalistic retrospective study, we examined 96 patients diagnosed with a depressive episode in accordance with DSM-5 who were treated with ECT at Charité—Universitätsmedizin Berlin. We studied change of depressive symptoms assessed with the MADRS during the course of ECT and tested, whether these could predict ECT outcome. Strongest reduction during the course of ECT and strongest predictive effects were found for affective symptoms, especially item 2 (reported sadness). Future longitudinal studies employing a variety of clinical interviews and self-report measures for depression severity are needed to validate our findings.

## Supplementary Information

Below is the link to the electronic supplementary material.

Supplementary file1 (DOCX 218 KB)

## Data Availability

The data that support the findings of this study are available from the corresponding author, LB, upon reasonable request.
