# Supplementary material for: A symptom-based approach in predicting ECT outcome in depressed patients employing MADRS single items
Source: Eur Arch Psychiatry Clin Neurosci. 2021 Jul 16;271(7):1275–84. doi: 10.1007/s00406-021-01301-8 (PMC8429160; doi:10.1007/s00406-021-01301-8)
Supplement: Supplementary file 1 — Supplementary file1 (DOCX 218 KB) [file 406_2021_1301_MOESM1_ESM.docx]

######

**A symptom-based approach in predicting ECT outcome in depressed patients employing MADRS single items**

Luisa Bönke^a^, Corinna Hartling^a^, Anna Stippl^a^, Ann-Kathrin Domke^a^, Ana Lucia Herrera-Mendelez^a^, Sabine Aust^a^, Matti Gärtner^a, c^, Malek Bajbouj^a^, Simone Grimm^a, b, c^

###### **Supplementary Material**

###### Table 2

*Frequencies of Clinical Characteristics*

| Variables | Frequencies |
| --- | --- |
| Diagnosis polarity | 86.5% unipolar (83)  13.5% bipolar (13) |
| Diagnosis | 3.1% F31.3: Bipolar affective disorder, current episode mild or moderate depression (3)  8.3% F31.4: Bipolar affective disorder, current episode severe depression without psychotic symptoms (8)  1.0% F31.5: Bipolar affective disorder, current episode severe depression with psychotic symptoms (1)  1.0% F31.6: Bipolar affective disorder, current episode mixed (1)  2.1% F32.1: Moderate depressive episode (2)  9.4% F32.2 Severe depressive episode without psychotic symptoms (9)  1.0% F32.3: Severe depressive episode with psychotic symptoms (1)  8.3% F33.1: Recurrent depressive disorder, current episode moderate (8)  53.1% F33.2: Recurrent depressive disorder, current episode severe without psychotic symptoms (51)  9.4% F33.3: Recurrent depressive disorder, current episode severe with psychotic symptoms (9)  3.1% F34.1: Dysthymia (3) |
| Psychiatric comorbidity | 46.9% yes (45)  53.1% no (51)  5.2% F00-F09: mental disorders due to known physiological disease (5)  16.7% F10-F19: present psychoactive substance use or dependence syndrome (16)  6.3% F10-F19: past psychoactive substance use or dependence syndrome (6)  3.1% F30-F39: affective disorder (3)  18.8% F40-F48: anxiety, stress-related, or somatoform disorders (18)  2.1% F50.-: eating disorders (2)  11.5% F60-F69: personality disorders (11)  1.0% F80-F89: pervasive developmental disorder (1)  1.0% F90-F98: behavioral and emotional disorders with onset occurring in childhood and adolescence (1) |
| Somatic disease | 83.3% yes (80) 16.7% no (16)  6.3% A00-B99: infectious or parasitic (6)  40.6% E00-E89: endocrine, nutritional or metabolic (39)  11.5% G00-G99: of the nervous system (11)  5.2% H00-H95: of the eye or ear or nose (5)  38.5% I00-I99: of the circulatory system and heart (37) 2.1% J00-J99: of the respiratory system (2) 11.5% K00-K95: of the digestive system (11)  17.7% L00-L99: of the skin or musculoskeletal system (17)  19.8% N00-N99: of the genitourinary system (19) |
|  | 11.5% Z00-Z99: presence of factors influencing health status (11)  8.3% G47.-: sleep disorder (8) |

*Note*. Census data in parentheses. Subgroups of psychiatric and somatic comorbidities refer to ICD-10 classifications. Percentage scores might not add up to exactly 100% due to patients having more than one diagnosis.

###### Table 3

*Frequencies of Antidepressant Medication Use*

| Variable | Frequencies |
| --- | --- |
| Antidepressants (ADs) at baseline | 32.3% none (31)  67.7% ADs (65):  9.4% SSRIs (9)  24.0% SSNRI (23) 10.4% NDRIs (10) 16.7% SARIs (16) 9.4% TCAs (9) 12.5% TeCAs (12) 2.1% MAOIs (2) |
| Other psychiatric medication | 28.1% none (27)  71.9% others (69):  56.3% antipsychotics (54)  25.0% mood stabilizer (24)  22.9% benzodiazepines (22) |
| Change in Medication | 19.8% no (19)  77.1% yes (74)  3.1% N/A (3) |
| Change in ADs | 49.0% no change (47)  31.3% change in type (30):  5.2% switch to different type (5)  9.4% cessation (9)  16.7% start of new AD (16)  16.7% change in dosage (16):  12.5% increase (12)  7.3% reduction (7)  3.1% N/A (3) |
| Change in other psychiatric medication | 36.5% no change (35)  32.3% change in type (31):  2.1% switch to different type (2)  19.8% cessation (19)  10.4% start of new medication (10)  28.1% change in dosage (27):  10.4% increase (10)  24.0% reduction (23)  3.1% N/A (3) |

*Note*. Census data in parentheses. SSRIs = selective Serotonin-Reuptake-Inhibitors. SSNRIs = selective Serotonin–norepinephrine reuptake inhibitors. Selective norepinephrine reuptake inhibitor. NDRIs = Norepinephrine-dopamine reuptake inhibitors. SARIs = Serotonin antagonist and reuptake inhibitors. TCAs = Tricyclic antidepressants. TeCAs = Tetracyclic antidepressants. MAOIs = Monoamine oxidase inhibitors. Percentage scores might not add up to exactly 100% due to patients taking more than one psychiatric medication.

###### Table 4

*Results for the stepwise logistic regression analysis, predicting response*

|  | Model 2 | | | | | | | | | | |
| --- | --- | --- | --- | --- | --- | --- | --- | --- | --- | --- | --- |
| Variables | ß | *Wald* | *df* | *p* | *OR* | *R*^2^ | *f* | χ² | *df* | *p* | % |
| Step 1: |  |  |  |  |  |  |  |  |  |  |  |
| **Gender** | **1.46** | **9.03** | **1** | **.003** | **4.32** |  |  |  |  |  |  |
| **Psychotic Symptoms** | **2.86** | **6.55** | **1** | **.010** | **17.40** |  |  |  |  |  |  |
| Number of ECT sessions | -0.13 | 2.37 | 1 | .124 | 0.88 |  |  |  |  |  |  |
|  |  |  | 1 |  |  | .25 | 0.58 | 20.11 | 3 | < .001 | 67.7 |
| Step 2: |  |  |  |  |  |  |  |  |  |  |  |
| **Gender** | **1.45** | **8.05** | **1** | **.005** | **4.26** |  |  |  |  |  |  |
| **Psychotic Symptoms** | **2.59** | **5.37** | **1** | **.020** | **13.28** |  |  |  |  |  |  |
| Number of ECT sessions | 1.07 | 2.98 | 1 | .084 | 2.92 |  |  |  |  |  |  |
| ECTs*MADRS T0 Total Score | -0.04 | 3.65 | 1 | .056 | 0.96 |  |  |  |  |  |  |
| **MADRS T0 Total Score** | **0.62** | **4.71** | **1** | **.030** | **1.88** |  |  |  |  |  |  |
|  |  |  |  |  |  | **.34** | **0.72** | **28.07** | **5** | **< .001** | **67.7** |
|  |  |  |  |  |  |  |  |  |  |  |  |
| **Gender** | **1.45** | **8.26** | **1** | **.004** | **4.27** |  |  |  |  |  |  |
| **Psychotic Symptoms** | **2.56** | **5.18** | **1** | **.023** | **12.99** |  |  |  |  |  |  |
| Number of ECT sessions | -0.10 | 1.16 | 1 | .282 | 0.91 |  |  |  |  |  |  |
| **MADRS T0 Item 1** | **0.65** | **4.78** | **1** | **.029** | **1.91** |  |  |  |  |  |  |
|  |  |  |  |  |  | **.31** | **0.67** | **25.54** | **4** | **< .001** | **68.8** |
|  |  |  |  |  |  |  |  |  |  |  |  |
| **Gender** | **10.59** | **6.60** | **1** | **.010** | **39753.94** |  |  |  |  |  |  |
| Psychotic Symptoms | 2.21 | 3.28 | 1 | .070 | 9.15 |  |  |  |  |  |  |
| Number of ECT sessions | -.141 | 2.46 | 1 | .117 | 0.87 |  |  |  |  |  |  |
| **Gender*MADRS T0 Item 2** | **-2.16** | **5.30** | **1** | **.021** | **0.12** |  |  |  |  |  |  |
| **MADRS T0 Item 2** | **4.41** | **6.28** | **1** | **.012** | **82.10** |  |  |  |  |  |  |
|  |  |  |  |  |  | **.37** | **0.77** | **31.60** | **5** | **< .001** | **69.8** |
|  |  |  |  |  |  |  |  |  |  |  |  |
| **Gender** | **1.47** | **9.03** | **1** | **.003** | **4.33** |  |  |  |  |  |  |
| **Psychotic Symptoms** | **2.87** | **6.61** | **1** | **.010** | **17.71** |  |  |  |  |  |  |
| Number of ECT sessions | -0.13 | 2.41 | 1 | .121 | 0.88 |  |  |  |  |  |  |
| MADRS T0 Item 3 | -0.07 | 0.09 | 1 | .767 | 0.93 |  |  |  |  |  |  |
|  |  |  |  |  |  | .25 | 0.58 | 20.19 | 4 | < .001 | 68.8 |
|  |  |  |  |  |  |  |  |  |  |  |  |
| **Gender** | **1.46** | **9.03** | **1** | **.003** | **4.32** |  |  |  |  |  |  |
| **Psychotic Symptoms** | **2.87** | **6.43** | **1** | **.011** | **17.55** |  |  |  |  |  |  |
| Number of ECT sessions | -0.13 | 2.33 | 1 | .127 | 0.88 |  |  |  |  |  |  |
| MADRS T0 Item 4 | -0.01 | .003 | 1 | .960 | 0.99 |  |  |  |  |  |  |
|  |  |  |  |  |  | .25 | 0.58 | 20.11 | 4 | < .001 | 67.7 |
|  |  |  |  |  |  |  |  |  |  |  |  |
| **Gender** | **1.44** | **8.678** | **1** | **.003** | **4.22** |  |  |  |  |  |  |
| **Psychotic Symptoms** | **2.93** | **6.690** | **1** | **.010** | **18.61** |  |  |  |  |  |  |
| Number of ECT sessions | -0.13 | 2.36 | 1 | .125 | 0.88 |  |  |  |  |  |  |
| MADRS T0 Item 5 | 0.11 | .404 | 1 | .525 | 1.11 |  |  |  |  |  |  |
|  |  |  |  |  |  | .26 | 0.59 | 20.51 | 4 | < .001 | 67.7 |
|  |  |  |  |  |  |  |  |  |  |  |  |
| **Gender** | **1.43** | **8.31** | **1** | **.004** | **4.19** |  |  |  |  |  |  |
| **Psychotic Symptoms** | **2.80** | **6.19** | **1** | **.013** | **16.51** |  |  |  |  |  |  |
| Number of ECT sessions | -0.13 | 2.29 | 1 | .131 | 0.88 |  |  |  |  |  |  |
| MADRS T0 Item 6 | 0.07 | 0.08 | 1 | .773 | 1.08 |  |  |  |  |  |  |
|  |  |  |  |  |  | .25 | 0.58 | 20.19 | 4 | < .001 | 67.7 |
|  |  |  |  |  |  |  |  |  |  |  |  |
| **Gender** | **1.55** | **9.61** | **1** | **.002** | **4.71** |  |  |  |  |  |  |
| **Psychotic Symptoms** | **2.97** | **6.47** | **1** | **.011** | **19.44** |  |  |  |  |  |  |
| Number of ECT sessions | -0.13 | 2.58 | 1 | .108 | 0.87 |  |  |  |  |  |  |
| MADRS T0 Item 7 | 0.31 | 1.54 | 1 | .215 | 1.36 |  |  |  |  |  |  |
|  |  |  |  |  |  | .27 | 0.61 | 21.66 | 4 | < .001 | 68.8 |
|  |  |  |  |  |  |  |  |  |  |  |  |
| **Gender** | **1.49** | **8.55** | **1** | **.003** | **4.42** |  |  |  |  |  |  |
| **Psychotic Symptoms** | **2.81** | **6.34** | **1** | **.012** | **16.53** |  |  |  |  |  |  |
| Number of ECT sessions | 0.45 | 2.29 | 1 | .131 | 1.57 |  |  |  |  |  |  |
| **ECTs* MADRS T0 Item 8** | **-.17** | **3.99** | **1** | **.046** | **0.84** |  |  |  |  |  |  |
| **MADRS T0 Item 8** | **2.59** | **4.67** | **1** | **.031** | **13.27** |  |  |  |  |  |  |
|  |  |  |  |  |  | **.32** | **0.69** | **26.33** | **5** | **< .001** | **71.9** |
|  |  |  |  |  |  |  |  |  |  |  |  |
| **Gender** | **1.45** | **8.57** | **1** | **.003** | **4.27** |  |  |  |  |  |  |
| **Psychotic Symptoms** | **2.86** | **5.44** | **1** | **.020** | **17.46** |  |  |  |  |  |  |
| Number of ECT sessions | 0.22 | 1.05 | 1 | .306 | 1.25 |  |  |  |  |  |  |
| ECTs* MADRS T0 Item 9 | -0.14 | 2.89 | 1 | .089 | 0.87 |  |  |  |  |  |  |
| MADRS T0 Item 9 | 2.04 | 3.40 | 1 | .065 | 7.68 |  |  |  |  |  |  |
|  |  |  |  |  |  | .30 | 0.65 | 24.27 | 5 | < .001 | 71.9 |
|  |  |  |  |  |  |  |  |  |  |  |  |
| **Gender** | **1.55** | **9.60** | **1** | **.002** | **4.71** |  |  |  |  |  |  |
| **Psychotic Symptoms** | **3.10** | **7.38** | **1** | **.007** | **22.29** |  |  |  |  |  |  |
| Number of ECT sessions | -0.11 | 1.63 | 1 | .202 | 0.90 |  |  |  |  |  |  |
| MADRS T0 Item 10 | 0.23 | 1.87 | 1 | .171 | 1.26 |  |  |  |  |  |  |
|  |  |  |  |  |  | .27 | 0.61 | 22.02 | 4 | < .001 | 69.8 |
|  |  |  |  |  |  |  |  |  |  |  |  |
| **Gender** | **1.29** | **5.37** | **1** | **.020** | **3.64** |  |  |  |  |  |  |
| **Psychotic Symptoms** | **3.16** | **6.17** | **1** | **.013** | **23.64** |  |  |  |  |  |  |
| Number of ECT sessions | **-**0.10 | 1.15 | 1 | .283 | 0.90 |  |  |  |  |  |  |
| **MADRS T1 Total Score*** | **-0.17** | **14.48** | **1** | **< .001** | **0.84** |  |  |  |  |  |  |
|  |  |  |  |  |  | **.45** | **0.90** | **39.79** | **4** | **< .001** | **76.0** |
|  |  |  |  |  |  |  |  |  |  |  |  |
| **Gender** | **1.53** | **8.60** | **1** | **.003** | **4.62** |  |  |  |  |  |  |
| **Psychotic Symptoms** | **2.96** | **6.46** | **1** | **.011** | **19.38** |  |  |  |  |  |  |
| Number of ECT sessions | -0.11 | 1.38 | 1 | .240 | 0.90 |  |  |  |  |  |  |
| **MADRS T1 Item 1*** | **-0.68** | **8.240** | **1** | **.004** | **0.51** |  |  |  |  |  |  |
|  |  |  |  |  |  | **.36** | **0.75** | **29.64** | **4** | **< .001** | **70.8** |
|  |  |  |  |  |  |  |  |  |  |  |  |
| **Gender** | **1.47** | **7.68** | **1** | **.006** | **4.33** |  |  |  |  |  |  |
| **Psychotic Symptoms** | **3.07** | **6.61** | **1** | **.010** | **21.48** |  |  |  |  |  |  |
| Number of ECT sessions | -0.11 | 1.55 | 1 | .213 | 0.89 |  |  |  |  |  |  |
| **MADRS T1 Item 2*** | **-0.67** | **8.83** | **1** | **.003** | **0.51** |  |  |  |  |  |  |
|  |  |  |  |  |  | **.37** | **0.77** | **30.64** | **4** | **< .001** | **78.1** |
|  |  |  |  |  |  |  |  |  |  |  |  |
| **Gender** | **1.43** | **7.64** | **1** | **.006** | **4.18** |  |  |  |  |  |  |
| **Psychotic Symptoms** | **3.22** | **7.25** | **1** | **.007** | **24.93** |  |  |  |  |  |  |
| Number of ECT sessions | -0.15 | 2.74 | 1 | .098 | 0.86 |  |  |  |  |  |  |
| **MADRS T1 Item 3** | **-0.64** | **7.93** | **1** | **.005** | **0.53** |  |  |  |  |  |  |
|  |  |  |  |  |  | **.35** | **0.73** | **29.12** | **4** | **< .001** | **68.8** |
|  |  |  |  |  |  |  |  |  |  |  |  |
| **Gender** | **1.38** | **7.61** | **1** | **.006** | **3.98** |  |  |  |  |  |  |
| **Psychotic Symptoms** | **3.00** | **6.80** | **1** | **.009** | **20.08** |  |  |  |  |  |  |
| Number of ECT sessions | -0.09 | 1.06 | 1 | .304 | 0.91 |  |  |  |  |  |  |
| **MADRS T1 Item 4** | **-0.50** | **4.70** | **1** | **.030** | **0.61** |  |  |  |  |  |  |
|  |  |  |  |  |  | **.31** | **0.67** | **25.25** | **4** | **< .001** | **70.8** |
|  |  |  |  |  |  |  |  |  |  |  |  |
| **Gender** | **1.48** | **8.67** | **1** | **.003** | **4.37** |  |  |  |  |  |  |
| **Psychotic Symptoms** | **2.53** | **5.04** | **1** | **.025** | **12.57** |  |  |  |  |  |  |
| Number of ECT sessions | -0.14 | 2.38 | 1 | .123 | 0.87 |  |  |  |  |  |  |
| **MADRS T1 Item 5** | **-0.45** | **4.75** | **1** | **.029** | **0.64** |  |  |  |  |  |  |
|  |  |  |  |  |  | **.31** | **0.67** | **25.34** | **4** | **< .001** | **72.9** |
|  |  |  |  |  |  |  |  |  |  |  |  |
| **Gender** | **1.54** | **9.09** | **1** | **.003** | **4.67** |  |  |  |  |  |  |
| **Psychotic Symptoms** | **3.01** | **6.68** | **1** | **.010** | **20.30** |  |  |  |  |  |  |
| Number of ECT sessions | -0.15 | 2.74 | 1 | .098 | 0.87 |  |  |  |  |  |  |
| **MADRS T1 Item 6** | **-0.52** | **5.41** | **1** | **.020** | **0.60** |  |  |  |  |  |  |
|  |  |  |  |  |  | **.32** | **0.69** | **25.99** | **4** | **< .001** | **76.0** |
|  |  |  |  |  |  |  |  |  |  |  |  |
| **Gender** | **1.41** | **7.77** | **1** | **.005** | **4.09** |  |  |  |  |  |  |
| **Psychotic Symptoms** | **2.78** | **5.86** | **1** | **.016** | **16.11** |  |  |  |  |  |  |
| Number of ECT sessions | -0.11 | 1.45 | 1 | .229 | 0.90 |  |  |  |  |  |  |
| **MADRS T1 Item 7** | **-0.58** | **5.53** | **1** | **.019** | **0.56** |  |  |  |  |  |  |
|  |  |  |  |  |  | **.32** | **0.69** | **26.29** | **4** | **< .001** | **71.9** |
|  |  |  |  |  |  |  |  |  |  |  |  |
| **Gender** | **1.33** | **6.87** | **1** | **.009** | **3.79** |  |  |  |  |  |  |
| **Psychotic Symptoms** | **2.94** | **6.33** | **1** | **.012** | **18.87** |  |  |  |  |  |  |
| Number of ECT sessions | -0.13 | 2.04 | 1 | .153 | 0.88 |  |  |  |  |  |  |
| **MADRS T1 Item 8** | **-0.57** | **6.81** | **1** | **.009** | **0.57** |  |  |  |  |  |  |
|  |  |  |  |  |  | **.34** | **0.72** | **27.77** | **4** | **< .001** | **75.0** |
|  |  |  |  |  |  |  |  |  |  |  |  |
| **Gender** | **1.21** | **5.57** | **1** | **.018** | **3.35** |  |  |  |  |  |  |
| **Psychotic Symptoms** | **3.13** | **6.81** | **1** | **.009** | **22.91** |  |  |  |  |  |  |
| Number of ECT sessions | -0.10 | 1.33 | 1 | .248 | 0.90 |  |  |  |  |  |  |
| **MADRS T1 Item 9** | **-0.55** | **6.44** | **1** | **.011** | **0.58** |  |  |  |  |  |  |
|  |  |  |  |  |  | **.33** | **0.70** | **27.18** | **4** | **< .001** | **71.9** |
|  |  |  |  |  |  |  |  |  |  |  |  |
| **Gender** | **1.41** | **7.80** | **1** | **.005** | **4.08** |  |  |  |  |  |  |
| **Psychotic Symptoms** | **2.62** | **5.42** | **1** | **.020** | **13.72** |  |  |  |  |  |  |
| Number of ECT sessions | -0.14 | 2.62 | 1 | .106 | 0.87 |  |  |  |  |  |  |
| **MADRS T1 Item 10** | **-0.51** | **4.39** | **1** | **.036** | **0.60** |  |  |  |  |  |  |
|  |  |  |  |  |  | **.31** | **0.67** | **25.01** | **4** | **< .001** | **69.8** |
|  |  |  |  |  |  |  |  |  |  |  |  |
| **Gender** | **1.17** | **3.92** | **1** | **.048** | **3.21** |  |  |  |  |  |  |
| **Psychotic Symptoms** | **2.63** | **4.62** | **1** | **.032** | **13.86** |  |  |  |  |  |  |
| Number of ECT sessions | -0.10 | 0.87 | 1 | .352 | 0.91 |  |  |  |  |  |  |
| **Change Score T0:T1 MADRS Total score*** | **-0.07** | **19.03** | **1** | **< .001** | **0.94** |  |  |  |  |  |  |
|  |  |  |  |  |  | **.55** | **1.11** | **50.56** | **4** | **< .001** | **80.2** |
|  |  |  |  |  |  |  |  |  |  |  |  |
| **Gender** | **1.49** | **7.03** | **1** | **.008** | **4.43** |  |  |  |  |  |  |
| **Psychotic Symptoms** | **2.39** | **4.33** | **1** | **.038** | **10.86** |  |  |  |  |  |  |
| Number of ECT sessions | -0.05 | 0.23 | 1 | .631 | 0.95 |  |  |  |  |  |  |
| **Change Score T0:T1 Item 1*** | **-0.04** | **15.02** | **1** | **< .001** | **0.96** |  |  |  |  |  |  |
|  |  |  |  |  |  | **.45** | **0.90** | **39.38** | **4** | **< .001** | **77.9** |
| Gender | 1.10 | 3.70 | 1 | .055 | 3.00 |  |  |  |  |  |  |
| Psychotic Symptoms | 2.17 | 3.45 | 1 | .063 | 8.79 |  |  |  |  |  |  |
| Number of ECT sessions | -0.11 | 1.13 | 1 | .287 | 0.90 |  |  |  |  |  |  |
| **Change Score T0:T1 Item 2*** | **-0.05** | **16.54** | **1** | **< .001** | **0.95** |  |  |  |  |  |  |
|  |  |  |  |  |  | **.49** | **0.99** | **44.08** | **4** | **< .001** | **80.2** |
|  |  |  |  |  |  |  |  |  |  |  |  |
| **Gender** | **2.09** | **9.13** | **1** | **.003** | **8.06** |  |  |  |  |  |  |
| **Psychotic Symptoms** | **3.45** | **6.66** | **1** | **.010** | **31.56** |  |  |  |  |  |  |
| Number of ECT sessions | -0.14 | 2.64 | 1 | .105 | 0.87 |  |  |  |  |  |  |
| Gender*Change Score T0:T1 Item 3 | 0.02 | 2.62 | 1 | .106 | 1.02 |  |  |  |  |  |  |
| **Change Score T0:T1 Item 3** | **-0.05** | **4.08** | **1** | **.044** | **0.95** |  |  |  |  |  |  |
|  |  |  |  |  |  | **.34** | **0.72** | **27.75** | **5** | **< .001** | **72.9** |
|  |  |  |  |  |  |  |  |  |  |  |  |
| **Gender** | **1.59** | **9.24** | **1** | **.002** | **4.89** |  |  |  |  |  |  |
| **Psychotic Symptoms** | **3.08** | **6.76** | **1** | **.009** | **21.73** |  |  |  |  |  |  |
| Number of ECT sessions | -0.12 | 1.80 | 1 | .179 | 0.88 |  |  |  |  |  |  |
| Change Score T0:T1 Item 4 | -0.01 | 2.11 | 1 | .146 | 0.99 |  |  |  |  |  |  |
|  |  |  |  |  |  | .32 | 0.69 | 24.03 | 4 | < .001 | 71.9 |
|  |  |  |  |  |  |  |  |  |  |  |  |
| **Gender** | **1.41** | **8.11** | **1** | **.004** | **4.09** |  |  |  |  |  |  |
| **Psychotic Symptoms** | **2.60** | **5.26** | **1** | **.022** | **13.51** |  |  |  |  |  |  |
| Number of ECT sessions | -0.14 | 2.57 | 1 | .109 | 0.87 |  |  |  |  |  |  |
| Change Score T0:T1 Item 5 | -0.00 | 1.68 | 1 | .195 | 1.00 |  |  |  |  |  |  |
|  |  |  |  |  |  | .25 | 0.58 | 19.04 | 4 | .001 | 66.7 |
|  |  |  |  |  |  |  |  |  |  |  |  |
| **Gender** | **1.44** | **7.03** | **1** | **.008** | **4.23** |  |  |  |  |  |  |
| **Psychotic Symptoms** | **2.74** | **5.68** | **1** | **.017** | **15.45** |  |  |  |  |  |  |
| Number of ECT sessions | -0.03 | 0.09 | 1 | .759 | 1.00 |  |  |  |  |  |  |
| ECTs*Change Score T0:T1 Item 6 | 0.01 | 3.51 | 1 | .061 | 1.01 |  |  |  |  |  |  |
| **Change Score T0:T1 Item 6** | **-1.00** | **5.30** | **1** | **.021** | **0.91** |  |  |  |  |  |  |
|  |  |  |  |  |  | **.41** | **0.83** | **34.81** | **4** | **< .001** | **70.5** |
|  |  |  |  |  |  |  |  |  |  |  |  |
| **Gender** | **1.55** | **9.17** | **1** | **.002** | **4.71** |  |  |  |  |  |  |
| **Psychotic Symptoms** | **2.58** | **5.22** | **1** | **.022** | **13.20** |  |  |  |  |  |  |
| Number of ECT sessions | -0.14 | 2.52 | 1 | .112 | 0.87 |  |  |  |  |  |  |
| **Change Score T0:T1 Item 7** | **-0.02** | **6.31** | **1** | **.012** | **0.98** |  |  |  |  |  |  |
|  |  |  |  |  |  | **.33** | **0.70** | **27.37** | **4** | **< .001** | **71.9** |
|  |  |  |  |  |  |  |  |  |  |  |  |
| **Gender** | **1.12** | **4.05** | **1** | **.044** | **3.06** |  |  |  |  |  |  |
| **Psychotic Symptoms** | **2.83** | **5.88** | **1** | **.015** | **16.93** |  |  |  |  |  |  |
| Number of ECT sessions | 0.15 | 1.04 | 1 | .309 | 1.16 |  |  |  |  |  |  |
| **ECTs* Change Score T0:T1 Item 8** | **0.01** | **7.41** | **1** | **.006** | **1.01** |  |  |  |  |  |  |
| **Change Score T0:T1 Item 8*** | **-0.13** | **9.20** | **1** | **.002** | **0.87** |  |  |  |  |  |  |
|  |  |  |  |  |  | **.48** | **0.96** | **42.29** | **5** | **< .001** | **77.1** |
|  |  |  |  |  |  |  |  |  |  |  |  |
| **Gender** | **1.25** | **6.02** | **1** | **.014** | **3.47** |  |  |  |  |  |  |
| **Psychotic Symptoms** | **2.44** | **4.61** | **1** | **.032** | **11.42** |  |  |  |  |  |  |
| Number of ECT sessions | -0.12 | 1.84 | 1 | .175 | 0.89 |  |  |  |  |  |  |
| **Change Score T0:T1 Item 9** | **-0.01** | **4.55** | **1** | **.033** | 0.99 |  |  |  |  |  |  |
|  |  |  |  |  |  | **.29** | **0.64** | **22.41** | **4** | **< .001** | **69.9** |
|  |  |  |  |  |  |  |  |  |  |  |  |
| **Gender** | **1.55** | **7.62** | **1** | **.006** | **4.72** |  |  |  |  |  |  |
| **Psychotic Symptoms** | **2.99** | **6.39** | **1** | **.011** | **19.79** |  |  |  |  |  |  |
| Number of ECT sessions | 0.19 | 1.85 | 1 | .174 | 1.20 |  |  |  |  |  |  |
| **ECTs* Change Score T0:T1 Item 10** | **0.01** | **6.95** | **1** | **.008** | **1.01** |  |  |  |  |  |  |
| **Change Score T0:T1 Item 10*** | **-1.00** | **9.34** | **1** | **.002** | **0.91** |  |  |  |  |  |  |
|  |  |  |  |  |  | **.43** | **0.87** | **35.47** | **5** | **< .001** | **78.0** |
|  |  |  |  |  |  |  |  |  |  |  |  |
| **Gender** | **1.45** | **5.38** | **1** | **.020** | **4.26** |  |  |  |  |  |  |
| Psychotic Symptoms | 1.89 | 2.52 | 1 | .113 | 6.63 |  |  |  |  |  |  |
| Number of ECT sessions | 0.01 | .01 | 1 | .946 | 1.01 |  |  |  |  |  |  |
| **Change Score T0:T2 Item 1*** | **-0.06** | **20.61** | **1** | **< .001** | **0.95** |  |  |  |  |  |  |
|  |  |  |  |  |  | **.57** | **1.15** | **53.19** | **4** | **< .001** | **83.2** |
|  |  |  |  |  |  |  |  |  |  |  |  |
| **Gender** | **1.89** | **3.90** | **1** | **.048** | **6.62** |  |  |  |  |  |  |
| Psychotic Symptoms | 2.02 | 2.68 | 1 | .102 | 7.51 |  |  |  |  |  |  |
| Number of ECT sessions | -0.16 | 1.16 | 1 | .281 | 0.85 |  |  |  |  |  |  |
| **Change Score T0:T2 Item 2*** | **-0.12** | **19.48** | **1** | **< .001** | **0.89** |  |  |  |  |  |  |
|  |  |  |  |  |  | **.81** | **2.06** | **89.42** | **4** | **< .001** | **91.7** |
|  |  |  |  |  |  |  |  |  |  |  |  |
| Gender | -1.61 | 1.50 | 1 | .221 | 0.20 |  |  |  |  |  |  |
| **Psychotic Symptoms** | **2.82** | **5.57** | **1** | **.018** | **16.78** |  |  |  |  |  |  |
| Number of ECT sessions | -0.19 | 3.08 | 1 | .079 | 0.83 |  |  |  |  |  |  |
| **Gender*Change Score T0:T2 Item 3** | **-0.06** | **6.79** | **1** | **.009** | **0.94** |  |  |  |  |  |  |
| Change Score T0:T2 Item 3 | 0.05 | 2.37 | 1 | .124 | 1.05 |  |  |  |  |  |  |
|  |  |  |  |  |  | .58 | 1.18 | 55.26 | 5 | < .001 | 83.3 |
|  |  |  |  |  |  |  |  |  |  |  |  |
| **Gender** | **1.30** | **6.54** | **1** | **.011** | **3.68** |  |  |  |  |  |  |
| **Psychotic Symptoms** | **2.67** | **5.61** | **1** | **.018** | **14.45** |  |  |  |  |  |  |
| Number of ECT sessions | -0.10 | 1.10 | 1 | .296 | 0.91 |  |  |  |  |  |  |
| **Change Score T0:T2 Item 4** | **-0.01** | **4.74** | **1** | **.030** | **0.99** |  |  |  |  |  |  |
|  |  |  |  |  |  | **.32** | **0.69** | **25.83** | **4** | **< .001** | **72.0** |
|  |  |  |  |  |  |  |  |  |  |  |  |
| **Gender** | **1.41** | **6.48** | **1** | **.011** | **4.08** |  |  |  |  |  |  |
| **Psychotic Symptoms** | **2.62** | **4.96** | **1** | **.026** | **13.71** |  |  |  |  |  |  |
| **Number of ECT sessions** | **-0.27** | **6.02** | **1** | **.014** | **0.76** |  |  |  |  |  |  |
| **Change Score T0:T2 Item 5*** | **-0.02** | **11.70** | **1** | **.001** | **0.98** |  |  |  |  |  |  |
|  |  |  |  |  |  | **.41** | **0.83** | **33.45** | **4** | **< .001** | **73.9** |
|  |  |  |  |  |  |  |  |  |  |  |  |
| **Gender** | **1.80** | **9.19** | **1** | **.002** | **6.02** |  |  |  |  |  |  |
| **Psychotic Symptoms** | **3.32** | **7.34** | **1** | **.007** | **27.66** |  |  |  |  |  |  |
| Number of ECT sessions | -0.13 | 1.96 | 1 | .162 | 0.88 |  |  |  |  |  |  |
| **Change Score T0:T2 Item 6*** | **-0.03** | **15.11** | **1** | **< .001** | **0.97** |  |  |  |  |  |  |
|  |  |  |  |  |  | **.49** | **0.99** | **43.21** | **4** | **< .001** | **80.0** |
|  |  |  |  |  |  |  |  |  |  |  |  |
| **Gender** | **1.39** | **6.00** | **1** | **.014** | **4.02** |  |  |  |  |  |  |
| **Psychotic Symptoms** | **3.61** | **3.74** | **1** | **.053** | **37.10** |  |  |  |  |  |  |
| **Number of ECT sessions** | **-0.46** | **3.95** | **1** | **.047** | **0.63** |  |  |  |  |  |  |
| ECTs*Change Score T0:T2 Item 7 | -0.01 | 3.10 | 1 | .078 | 0.99 |  |  |  |  |  |  |
| Change Score T0:T2 Item 7 | 0.06 | 1.15 | 1 | .283 | 1.06 |  |  |  |  |  |  |
|  |  |  |  |  |  | .53 | 1.06 | 48.50 | 5 | < .001 | 83.3 |
|  |  |  |  |  |  |  |  |  |  |  |  |
| **Gender** | **1.37** | **4.59** | **1** | **.032** | **3.95** |  |  |  |  |  |  |
| Psychotic Symptoms | 2.22 | 3.56 | 1 | .059 | 9.24 |  |  |  |  |  |  |
| Number of ECT sessions | -0.15 | 1.84 | 1 | .175 | 0.86 |  |  |  |  |  |  |
| **Change Score T0:T2 Item 8*** | **-0.06** | **18.23** | **1** | **< .001** | **0.94** |  |  |  |  |  |  |
|  |  |  |  |  |  | **.65** | **1.36** | **63.67** | **4** | **< .001** | **84.4** |
|  |  |  |  |  |  |  |  |  |  |  |  |
| **Gender** | **1.80** | **6.71** | **1** | **.010** | **6.07** |  |  |  |  |  |  |
| Psychotic Symptoms | 0.91 | 0.53 | 1 | .467 | 2.47 |  |  |  |  |  |  |
| **Number of ECT sessions** | **-0.22** | **3.85** | **1** | **.050** | **0.80** |  |  |  |  |  |  |
| **Change Score T0:T2 Item 9*** | **-0.05** | **17.39** | **1** | **< .001** | **0.95** |  |  |  |  |  |  |
|  |  |  |  |  |  | **.62** | **1.28** | **58.28** | **4** | **< .001** | **81.7** |
|  |  |  |  |  |  |  |  |  |  |  |  |
| **Gender** | **1.46** | **7.17** | **1** | **.007** | **4.31** |  |  |  |  |  |  |
| **Psychotic Symptoms** | **3.28** | **7.18** | **1** | **.007** | **26.56** |  |  |  |  |  |  |
| Number of ECT sessions | -0.03 | 0.07 | 1 | .788 | 0.97 |  |  |  |  |  |  |
| **Change Score T0:T2 Item 10*** | **-0.02** | **13.86** | **1** | **< .001** | **0.98** |  |  |  |  |  |  |
|  |  |  |  |  |  | **.45** | **0.90** | **38.67** | **4** | **< .001** | **76.3** |
|  |  |  |  |  |  |  |  |  |  |  |  |

*Note*. *R*^2^ = Nagelkerkes *R*^2^. % = Percentage of correct predicition. ECTs = Number of ECTs. Significant predictors and models including significant MADRS predictors are indicated in bold. *Bonferroni-corrected significance. *f* = Cohen’s *f.*

###### Table 5

*Results for the stepwise logistic regression analysis, predicting early response*

|  | Model 2 | | | | | | | | | | |
| --- | --- | --- | --- | --- | --- | --- | --- | --- | --- | --- | --- |
| Variables | ß | *Wald* | *df* | *p* | *OR* | *R*^2^ | *f* | χ² | *df* | *p* | % |
| Step 1: |  |  |  |  |  |  |  |  |  |  |  |
| Gender | 1.06 | 3.32 | 1 | .069 | 2.88 |  |  |  |  |  |  |
| **Psychotic Symptoms** | **1.84** | **6.66** | **1** | **.010** | **6.29** |  |  |  |  |  |  |
|  |  |  |  |  |  | .14 | 0.40 | 9.20 | 2 | .010 | 49.0 |
| Step 2: |  |  |  |  |  |  |  |  |  |  |  |
| Gender | 1.14 | 3.54 | 1 | .060 | 3.12 |  |  |  |  |  |  |
| **Psychotic Symptoms** | **1.78** | **6.17** | **1** | **.013** | **5.91** |  |  |  |  |  |  |
| MADRS T0 Total Score | 0.09 | 3.14 | 1 | .076 | 1.09 |  |  |  |  |  |  |
|  |  |  |  |  |  | .18 | 0.47 | 12.50 | 3 | .006 | 66.7 |
|  |  |  |  |  |  |  |  |  |  |  |  |
| Gender | 1.06 | 3.31 | 1 | .069 | 2.88 |  |  |  |  |  |  |
| **Psychotic Symptoms** | **1.82** | **6.27** | **1** | **.012** | **6.19** |  |  |  |  |  |  |
| MADRS T0 Item 1 | 0.03 | 0.01 | 1 | .916 | 1.03 |  |  |  |  |  |  |
|  |  |  |  |  |  | .14 | 0.40 | 9.21 | 3 | .027 | 49.0 |
|  |  |  |  |  |  |  |  |  |  |  |  |
| Gender | 1.05 | 3.26 | 1 | .071 | 2.87 |  |  |  |  |  |  |
| **Psychotic Symptoms** | **1.80** | **6.30** | **1** | **.012** | **6.02** |  |  |  |  |  |  |
| MADRS T0 Item 2 | 0.12 | 0.15 | 1 | .696 | 1.12 |  |  |  |  |  |  |
|  |  |  |  |  |  | .14 | 0.40 | 9.36 | 3 | .025 | 49.0 |
|  |  |  |  |  |  |  |  |  |  |  |  |
| Gender | 1.08 | 3.41 | 1 | .065 | 2.95 |  |  |  |  |  |  |
| **Psychotic Symptoms** | **1.80** | **6.36** | **1** | **.012** | **6.08** |  |  |  |  |  |  |
| MADRS T0 Item 3 | 0.28 | 1.01 | 1 | .314 | 1.33 |  |  |  |  |  |  |
|  |  |  |  |  |  | .15 | 0.42 | 10.25 | 3 | .017 | 65.6 |
|  |  |  |  |  |  |  |  |  |  |  |  |
| Gender | 1.06 | 3.32 | 1 | .069 | 2.88 |  |  |  |  |  |  |
| **Psychotic Symptoms** | **1.94** | **6.95** | **1** | **.008** | **6.98** |  |  |  |  |  |  |
| MADRS T0 Item 4 | -0.12 | 0.36 | 1 | .550 | 0.88 |  |  |  |  |  |  |
|  |  |  |  |  |  | .14 | 0.40 | 9.56 | 3 | .023 | 54.2 |
|  |  |  |  |  |  |  |  |  |  |  |  |
| Gender | 0.97 | 2.72 | 1 | .099 | 2.63 |  |  |  |  |  |  |
| **Psychotic Symptoms** | **2.00** | **7.16** | **1** | **.007** | **7.38** |  |  |  |  |  |  |
| MADRS T0 Item 5 | 0.31 | 2.87 | 1 | .090 | 1.36 |  |  |  |  |  |  |
|  |  |  |  |  |  | .18 | 0.47 | 12.17 | 3 | .007 | 60.9 |
|  |  |  |  |  |  |  |  |  |  |  |  |
| Gender | 1.05 | 3.20 | 1 | .074 | 2.87 |  |  |  |  |  |  |
| **Psychotic Symptoms** | **1.77** | **6.17** | **1** | **.013** | **5.84** |  |  |  |  |  |  |
| MADRS T0 Item 6 | 0.23 | 0.56 | 1 | .456 | 1.25 |  |  |  |  |  |  |
|  |  |  |  |  |  | .15 | 0.42 | 9.78 | 3 | .020 | 59.4 |
|  |  |  |  |  |  |  |  |  |  |  |  |
| Gender | 1.07 | 3.37 | 1 | .066 | 2.91 |  |  |  |  |  |  |
| **Psychotic Symptoms** | **1.83** | **6.53** | **1** | **.011** | **6.22** |  |  |  |  |  |  |
| MADRS T0 Item 7 | 0.08 | 0.10 | 1 | .758 | 1.09 |  |  |  |  |  |  |
|  |  |  |  |  |  | .14 | 0.40 | 9.30 | 3 | .026 | 55.2 |
|  |  |  |  |  |  |  |  |  |  |  |  |
| Gender | 1.15 | 3.66 | 1 | .056 | 3.15 |  |  |  |  |  |  |
| **Psychotic Symptoms** | **1.98** | **7.49** | **1** | **.006** | **7.26** |  |  |  |  |  |  |
| MADRS T0 Item 8 | 0.43 | 2.72 | 1 | .099 | 1.54 |  |  |  |  |  |  |
|  |  |  |  |  |  | .18 | 0.47 | 12.14 | 3 | .007 | 67.7 |
|  |  |  |  |  |  |  |  |  |  |  |  |
| Gender | 1.13 | 3.57 | 1 | .059 | 3.10 |  |  |  |  |  |  |
| **Psychotic Symptoms** | **1.78** | **6.30** | **1** | **.012** | **5.93** |  |  |  |  |  |  |
| MADRS T0 Item 9 | 0.17 | 0.71 | 1 | .401 | 1.18 |  |  |  |  |  |  |
|  |  |  |  |  |  | .15 | 0.42 | 9.91 | 3 | .019 | 69.8 |
|  |  |  |  |  |  |  |  |  |  |  |  |
| **Gender** | **1.16** | **3.84** | **1** | **.050** | **3.20** |  |  |  |  |  |  |
| **Psychotic Symptoms** | **2.22** | **8.37** | **1** | **.004** | **9.25** |  |  |  |  |  |  |
| MADRS T0 Item 10 | 0.34 | 3.44 | 1 | .064 | 1.40 |  |  |  |  |  |  |
|  |  |  |  |  |  | .19 | 0.48 | 12.66 | 3 | .005 | 70.8 |
|  |  |  |  |  |  |  |  |  |  |  |  |

*Note.* *R*^2^ = Nagelkerkes *R*^2^. % = Percentage of correct predicition. ECTs = Number of ECTs.

Significant predictors and models including significant MADRS predictors are indicated in bold.

*f* = Cohen’s *f.*

###### Table 6

*Results for the stepwise logistic regression analysis, predicting remission*

|  | Model 2 | | | | | | | | | | | |
| --- | --- | --- | --- | --- | --- | --- | --- | --- | --- | --- | --- | --- |
| Variables | ß | *Wald* | *df* | | *p* | *OR* | *R*^2^ | *f* | χ² | *df* | *p* | % |
| Step 1: |  |  |  |  | |  |  |  |  |  |  |  |
| **Gender** | **1.60** | **8.57** | **1** | **.003** | | **4.97** |  |  |  |  |  |  |
| **Psychotic Symptoms** | **1.44** | **3.96** | **1** | **.047** | | **4.24** |  |  |  |  |  |  |
| **Number of ECT sessions** | **-0.23** | **5.76** | **1** | **.016** | | **0.79** |  |  |  |  |  |  |
|  |  |  | 1 |  | |  | .23 | 0.55 | 17.48 | 3 | .001 | 68.8 |
| Step 2: |  |  | 1 |  | |  |  |  |  |  |  |  |
| **Gender** | **1.61** | **8.46** | **1** | **.004** | | **4.98** |  |  |  |  |  |  |
| Psychotic Symptoms | 1.35 | 3.52 | 1 | .061 | | 3.87 |  |  |  |  |  |  |
| **Number of ECT sessions** | **-0.22** | **5.03** | **1** | **.025** | | **0.80** |  |  |  |  |  |  |
| MADRS T0 Total Score | 0.04 | 0.89 |  | .347 | | 1.05 |  |  |  |  |  |  |
|  |  |  |  |  | |  | **.24** | **0.56** | **18.38** | **4** | **.001** | **66.7** |
|  |  |  |  |  | |  |  |  |  |  |  |  |
| **Gender** | **1.60** | **8.54** | **1** | **.003** | | **4.97** |  |  |  |  |  |  |
| Psychotic Symptoms | 1.41 | 3.60 | 1 | .058 | | 4.07 |  |  |  |  |  |  |
| **Number of ECT sessions** | **-0.23** | **5.37** | **1** | **.020** | | **0.80** |  |  |  |  |  |  |
| MADRS T0 Item 1 | 0.07 | 0.06 | 1 | .806 | | 1.07 |  |  |  |  |  |  |
|  |  |  |  |  | |  | .23 | 0.55 | 17.54 | 4 | .002 | 68.8 |
|  |  |  |  |  | |  |  |  |  |  |  |  |
| **Gender** | **1.59** | **8.26** | **1** | **.004** | | **4.90** |  |  |  |  |  |  |
| Psychotic Symptoms | 1.35 | 3.44 | 1 | .064 | | 3.84 |  |  |  |  |  |  |
| **Number of ECT sessions** | **-0.23** | **5.58** | **1** | **.018** | | **0.80** |  |  |  |  |  |  |
| MADRS T0 Item 2 | 0.21 | 0.56 | 1 | .456 | | 1.24 |  |  |  |  |  |  |
|  |  |  |  |  | |  | .24 | 0.56 | 18.06 | 4 | .001 | 65.6 |
|  |  |  |  |  | |  |  |  |  |  |  |  |
| **Gender** | **1.60** | **8.57** | **1** | **.003** | | **4.97** |  |  |  |  |  |  |
| **Psychotic Symptoms** | **1.44** | **3.89** | **1** | **.049** | | **4.21** |  |  |  |  |  |  |
| **Number of ECT sessions** | **-0.23** | **5.60** | **1** | **.018** | | **0.79** |  |  |  |  |  |  |
| MADRS T0 Item 3 | 0.03 | 0.02 | 1 | .900 | | 1.03 |  |  |  |  |  |  |
|  |  |  |  |  | |  | .23 | 0.55 | 17.50 | 4 | .002 | 68.8 |
|  |  |  |  |  | |  |  |  |  |  |  |  |
|  |  |  |  |  | |  |  |  |  |  |  |  |
| **Gender** | **1.60** | **8.52** | **1** | **.004** | | **4.93** |  |  |  |  |  |  |
| Psychotic Symptoms | 1.35 | 3.36 | 1 | .067 | | 3.86 |  |  |  |  |  |  |
| **Number of ECT sessions** | **-0.24** | **5.98** | **1** | **.014** | | **0.79** |  |  |  |  |  |  |
| MADRS T0 Item 4 | 0.12 | 0.41 | 1 | .525 | | 1.13 |  |  |  |  |  |  |
|  |  |  |  |  | |  | .24 | 0.56 | 17.89 | 4 | .001 | 67.7 |
|  |  |  |  |  | |  |  |  |  |  |  |  |
| **Gender** | **1.57** | **8.15** | **1** | **.004** | | **4.79** |  |  |  |  |  |  |
| **Psychotic Symptoms** | **1.47** | **4.05** | **1** | **.044** | | **4.34** |  |  |  |  |  |  |
| **Number of ECT sessions** | **-0.24** | **5.76** | **1** | **.016** | | **0.79** |  |  |  |  |  |  |
| MADRS T0 Item 5 | 0.11 | 0.43 | 1 | .513 | | 1.12 |  |  |  |  |  |  |
|  |  |  |  |  | |  | .24 | 0.56 | 17.91 | 4 | .001 | 67.7 |
|  |  |  |  |  | |  |  |  |  |  |  |  |
| **Gender** | **1.63** | **8.73** | **1** | **.003** | | **5.09** |  |  |  |  |  |  |
| **Psychotic Symptoms** | **1.50** | **4.03** | **1** | **.045** | | **4.49** |  |  |  |  |  |  |
| **Number of ECT sessions** | **-0.24** | **5.86** | **1** | **.016** | | 0.79 |  |  |  |  |  |  |
| MADRS T0 Item 6 | -0.11 | 0.16 | 1 | .686 | | 0.90 |  |  |  |  |  |  |
|  |  |  |  |  | |  | .23 | 0.55 | 17.64 | 4 | .001 | 67.7 |
|  |  |  |  |  | |  |  |  |  |  |  |  |
| **Gender** | **1.63** | **8.69** | **1** | **.003** | | **5.10** |  |  |  |  |  |  |
| **Psychotic Symptoms** | **1.42** | **3.77** | **1** | **.052** | | **4.13** |  |  |  |  |  |  |
| **Number of ECT sessions** | **-0.24** | **5.83** | **1** | **.016** | | **0.79** |  |  |  |  |  |  |
| MADRS T0 Item 7 | 0.11 | 0.19 | 1 | .666 | | 1.12 |  |  |  |  |  |  |
|  |  |  |  |  | |  | .23 | 0.55 | 17.66 | 4 | .001 | 66.7 |
|  |  |  |  |  | |  |  |  |  |  |  |  |
| **Gender** | **1.62** | **8.60** | **1** | **.003** | | **5.06** |  |  |  |  |  |  |
| **Psychotic Symptoms** | **1.47** | **4.13** | **1** | **.042** | | **4.38** |  |  |  |  |  |  |
| **Number of ECT sessions** | **-0.22** | **5.10** | **1** | **.024** | | **0.80** |  |  |  |  |  |  |
| MADRS T0 Item 8 | 0.21 | 0.86 | 1 | .354 | | 1.24 |  |  |  |  |  |  |
|  |  |  |  |  | |  | .24 | 0.56 | 18.35 | 4 | .001 | 66.7 |
|  |  |  |  |  | |  |  |  |  |  |  |  |
| **Gender** | **1.69** | **8.94** | **1** | **.003** | | **5.43** |  |  |  |  |  |  |
| Psychotic Symptoms | 1.34 | 3.43 | 1 | .064 | | 3.82 |  |  |  |  |  |  |
| **Number of ECT sessions** | **-0.22** | **4.99** | **1** | **.025** | | **0.80** |  |  |  |  |  |  |
| MADRS T0 Item 9 | 0.26 | 1.72 | 1 | .190 | | 1.30 |  |  |  |  |  |  |
|  |  |  |  |  | |  | .25 | 0.58 | 19.26 | 4 | .001 | 66.7 |
|  |  |  |  |  | |  |  |  |  |  |  |  |
| **Gender** | **1.60** | **8.53** | **1** | **.003** | | **4.97** |  |  |  |  |  |  |
| Psychotic Symptoms | 1.40 | 3.62 | 1 | .057 | | 4.07 |  |  |  |  |  |  |
| **Number of ECT sessions** | **-0.24** | **5.83** | **1** | **.016** | | **0.79** |  |  |  |  |  |  |
| MADRS T0 Item 10 | -0.06 | .12 | 1 | .732 | | 0.94 |  |  |  |  |  |  |
|  |  |  |  |  | |  | .23 | 0.55 | 17.60 | 4 | .001 | 67.7 |
|  |  |  |  |  | |  |  |  |  |  |  |  |
| **Gender** | **1.33** | **5.30** | **1** | **.021** | | **3.80** |  |  |  |  |  |  |
| Psychotic Symptoms | 1.08 | 1.79 | 1 | .181 | | 2.95 |  |  |  |  |  |  |
| **Number of ECT sessions** | **-0.21** | **4.02** | **1** | **.045** | | 0.81 |  |  |  |  |  |  |
| **MADRS T1 Total Score*** | **-0.12** | **9.23** | **1** | **.002** | | 0.89 |  |  |  |  |  |  |
|  |  |  |  |  | |  | **.36** | **0.75** | **28.55** | **4** | **< .001** | **70.8** |
|  |  |  |  |  | |  |  |  |  |  |  |  |
| **Gender** | **1.57** | **7.67** | **1** | **.006** | | **4.80** |  |  |  |  |  |  |
| Psychotic Symptoms | 1.30 | 2.86 | 1 | .091 | | 3.66 |  |  |  |  |  |  |
| **Number of ECT sessions** | **-0.21** | **4.50** | **1** | **.034** | | **0.81** |  |  |  |  |  |  |
| **MADRS T1 Item 1** | **-0.58** | **6.03** | **1** | **.014** | | **0.56** |  |  |  |  |  |  |
|  |  |  |  |  | |  | **.31** | **0.67** | **24.25** | **4** | **< .001** | **68.8** |
|  |  |  |  |  | |  |  |  |  |  |  |  |
| **Gender** | **1.50** | **6.68** | **1** | **.010** | | **4.49** |  |  |  |  |  |  |
| Psychotic Symptoms | 1.28 | 2.49 | 1 | .115 | | 3.59 |  |  |  |  |  |  |
| **Number of ECT sessions** | **-0.23** | **4.65** | **1** | **.031** | | **0.80** |  |  |  |  |  |  |
| **MADRS T1 Item 2*** | **-0.67** | **9.41** | **1** | **.002** | | **0.51** |  |  |  |  |  |  |
|  |  |  |  |  | |  | **.35** | **0.73** | **28.30** | **4** | **< .001** | **68.8** |
|  |  |  |  |  | |  |  |  |  |  |  |  |
| **Gender** | **1.58** | **7.29** | **1** | **.007** | | **4.84** |  |  |  |  |  |  |
| **Psychotic Symptoms** | **1.57** | **3.84** | **1** | **.050** | | **4.78** |  |  |  |  |  |  |
| **Number of ECT sessions** | **-0.26** | **6.22** | **1** | **.013** | | **0.77** |  |  |  |  |  |  |
| **MADRS T1 Item 3*** | **-0.72** | **9.21** | **1** | **.002** | | **0.49** |  |  |  |  |  |  |
|  |  |  |  |  | |  | **.35** | **0.73** | **28.21** | **4** | **< .001** | **70.8** |
|  |  |  |  |  | |  |  |  |  |  |  |  |
| **Gender** | **1.53** | **7.64** | **1** | **.006** | | **4.61** |  |  |  |  |  |  |
| **Psychotic Symptoms** | **1.42** | **3.73** | **1** | **.053** | | **4.14** |  |  |  |  |  |  |
| **Number of ECT sessions** | **-0.22** | **4.83** | **1** | **.028** | | **0.81** |  |  |  |  |  |  |
| MADRS T1 Item 4 | -0.19 | 0.73 | 1 | .394 | | 0.82 |  |  |  |  |  |  |
|  |  |  |  |  | |  | .24 | 0.56 | 18.22 | 4 | .001 | 67.7 |
|  |  |  |  |  | |  |  |  |  |  |  |  |
| **Gender** | **1.55** | **7.92** | **1** | **.005** | | **4.69** |  |  |  |  |  |  |
| Psychotic Symptoms | 1.16 | 2.42 | 1 | .120 | | 3.19 |  |  |  |  |  |  |
| **Number of ECT sessions** | **-0.23** | **5.54** | **1** | **.019** | | **0.79** |  |  |  |  |  |  |
| MADRS T1 Item 5 | -0.35 | 2.35 | 1 | .125 | | 0.71 |  |  |  |  |  |  |
|  |  |  |  |  | |  | .26 | 0.59 | 20.04 | 4 | < .001 | 72.9 |
|  |  |  |  |  | |  |  |  |  |  |  |  |
| **Gender** | **1.57** | **8.23** | **1** | **.004** | | **4.81** |  |  |  |  |  |  |
| Psychotic Symptoms | 1.38 | 3.58 | 1 | .059 | | 3.99 |  |  |  |  |  |  |
| **Number of ECT sessions** | **-0.24** | **5.88** | **1** | **.015** | | **0.79** |  |  |  |  |  |  |
| MADRS T1 Item 6 | -0.21 | 0.99 | 1 | .319 | | 0.81 |  |  |  |  |  |  |
|  |  |  |  |  | |  | .24 | 0.56 | 18.45 | 4 | .001 | 67.7 |
|  |  |  |  |  | |  |  |  |  |  |  |  |
| **Gender** | **1.48** | **7.16** | **1** | **.007** | | **4.38** |  |  |  |  |  |  |
| Psychotic Symptoms | 1.28 | 2.97 | 1 | .085 | | 3.61 |  |  |  |  |  |  |
| **Number of ECT sessions** | **-0.22** | **4.92** | **1** | **.027** | | **0.80** |  |  |  |  |  |  |
| MADRS T1 Item 7 | -0.42 | 2.99 | 1 | .084 | | 0.66 |  |  |  |  |  |  |
|  |  |  |  |  | |  | .27 | 0.61 | 20.65 | 4 | < .001 | 69.8 |
|  |  |  |  |  | |  |  |  |  |  |  |  |
| **Gender** | **1.41** | **6.26** | **1** | **.012** | | **4.10** |  |  |  |  |  |  |
| Psychotic Symptoms | 1.29 | 2.87 | 1 | .091 | | 3.63 |  |  |  |  |  |  |
| **Number of ECT sessions** | **-0.22** | **5.06** | **1** | **.024** | | **0.80** |  |  |  |  |  |  |
| **MADRS T1 Item 8** | **-0.61** | **6.44** | **1** | **.011** | | **0.54** |  |  |  |  |  |  |
|  |  |  |  |  | |  | **.32** | **0.69** | **24.97** | **4** | **< .001** | **69.8** |
|  |  |  |  |  | |  |  |  |  |  |  |  |
| **Gender** | **1.39** | **6.07** | **1** | **.014** | | **3.99** |  |  |  |  |  |  |
| Psychotic Symptoms | 1.38 | 3.40 | 1 | .065 | | 3.96 |  |  |  |  |  |  |
| **Number of ECT sessions** | **-0.21** | **4.50** | **1** | **.034** | | **0.81** |  |  |  |  |  |  |
| MADRS T1 Item 9 | -0.41 | 3.56 | 1 | .059 | | 0.66 |  |  |  |  |  |  |
|  |  |  |  |  | |  | .27 | 0.61 | 21.26 | 4 | < .001 | 68.8 |
|  |  |  |  |  | |  |  |  |  |  |  |  |
| **Gender** | **1.57** | **7.98** | **1** | **.005** | | **4.80** |  |  |  |  |  |  |
| Psychotic Symptoms | 1.30 | 3.07 | 1 | .080 | | 3.66 |  |  |  |  |  |  |
| **Number of ECT sessions** | **-0.24** | **5.92** | **1** | **.015** | | **0.79** |  |  |  |  |  |  |
| MADRS T1 Item 10 | -0.29 | 1.43 | 1 | .231 | | 0.75 |  |  |  |  |  |  |
|  |  |  |  |  | |  | .25 | 0.58 | 19.01 | 4 | .001 | 66.7 |
|  |  |  |  |  | |  |  |  |  |  |  |  |
| **Gender** | **1.26** | **4.46** | **1** | **.035** | | **3.52** |  |  |  |  |  |  |
| Psychotic Symptoms | 0.86 | 1.15 | 1 | .284 | | 2.36 |  |  |  |  |  |  |
| Number of ECT sessions | -0.19 | 3.20 | 1 | .074 | | 0.83 |  |  |  |  |  |  |
| **Change Score T0:T1 MADRS Total Score*** | **-0.04** | **11.77** | **1** | **.001** | | **0.96** |  |  |  |  |  |  |
|  |  |  |  |  | |  | **.39** | **0.64** | **32.10** | **4** | **< .001** | **75.0** |
|  |  |  |  |  | |  |  |  |  |  |  |  |
| **Gender** | **1.55** | **7.09** | **1** | **.008** | | **4.71** |  |  |  |  |  |  |
| Psychotic Symptoms | 1.03 | 1.82 | 1 | .178 | | 2.81 |  |  |  |  |  |  |
| Number of ECT sessions | -0.18 | 3.17 | 1 | .075 | | 0.83 |  |  |  |  |  |  |
| **Change Score T0:T1 Item 1** | **-0.03** | **8.02** | **1** | **.005** | | **0.97** |  |  |  |  |  |  |
|  |  |  |  |  | |  | **.33** | **0.80** | **25.97** | **4** | **< .001** | **68.4** |
|  |  |  |  |  | |  |  |  |  |  |  |  |
| **Gender** | **1.26** | **4.27** | **1** | **.039** | | **3.51** |  |  |  |  |  |  |
| Psychotic Symptoms | 0.84 | 1.15 | 1 | .283 | | 2.31 |  |  |  |  |  |  |
| **Number of ECT sessions** | **-0.21** | **3.84** | **1** | **.050** | | **0.81** |  |  |  |  |  |  |
| **Change Score T0:T1 Item 2*** | **-0.04** | **13.26** | **1** | **< .001** | | **0.96** |  |  |  |  |  |  |
|  |  |  |  |  | |  | **.42** | **0.70** | **34.64** | **4** | **< .001** | **68.8** |
|  |  |  |  |  | |  |  |  |  |  |  |  |
| **Gender** | **1.59** | **7.63** | **1** | **.006** | | **4.92** |  |  |  |  |  |  |
| Psychotic Symptoms | 1.49 | 3.57 | 1 | .059 | | 4.42 |  |  |  |  |  |  |
| **Number of ECT sessions** | **-0.23** | **5.29** | **1** | **.021** | | **0.80** |  |  |  |  |  |  |
| **Change Score T0:T1 Item 3** | **-0.02** | **6.78** | **1** | **.009** | | **0.98** |  |  |  |  |  |  |
|  |  |  |  |  | |  | **.33** | **0.85** | **25.71** | **4** | **< .001** | **70.8** |
|  |  |  |  |  | |  |  |  |  |  |  |  |
| **Gender** | **1.49** | **7.02** | **1** | **.008** | | **4.45** |  |  |  |  |  |  |
| Psychotic Symptoms | 1.38 | 3.55 | 1 | .059 | | 3.98 |  |  |  |  |  |  |
| **Number of ECT sessions** | **-0.23** | **5.34** | **1** | **.021** | | **0.79** |  |  |  |  |  |  |
| Change Score T0:T1 Item 4 | 0.00 | 0.36 | 1 | .548 | | 1.00 |  |  |  |  |  |  |
|  |  |  |  |  | |  | .24 | 0.70 | 17.10 | 4 | .002 | 67.4 |
|  |  |  |  |  | |  |  |  |  |  |  |  |
| **Gender** | **1.61** | **8.27** | **1** | **.004** | | **4.98** |  |  |  |  |  |  |
| Psychotic Symptoms | 1.11 | 2.16 | 1 | .142 | | 3.04 |  |  |  |  |  |  |
| **Number of ECT sessions** | **-0.23** | **5.52** | **1** | **.019** | | **0.79** |  |  |  |  |  |  |
| Change Score T0:T1 Item 5 | 0.00 | 0.39 | 1 | .535 | | 1.00 |  |  |  |  |  |  |
|  |  |  |  |  | |  | .22 | 0.56 | 16.28 | 4 | .003 | 66.7 |
|  |  |  |  |  | |  |  |  |  |  |  |  |
| **Gender** | **1.87** | **8.70** | **1** | **.003** | | **6.50** |  |  |  |  |  |  |
| **Psychotic Symptoms** | **1.57** | **3.97** | **1** | **.046** | | **4.79** |  |  |  |  |  |  |
| Number of ECT sessions | -0.12 | 1.17 | 1 | .279 | | 0.89 |  |  |  |  |  |  |
| **ECTs* Change Score T0:T1 Item 6** | **0.01** | **4.32** |  | **.038** | | **1.01** |  |  |  |  |  |  |
| **Change Score T0:T1 Item 6** | **-0.08** | **5.05** | **1** | **.025** | | **0.92** |  |  |  |  |  |  |
|  |  |  |  |  | |  | **.33** | **0.53** | **25.45** | **5** | **< .001** | **69.5** |
|  |  |  |  |  | |  |  |  |  |  |  |  |
| **Gender** | **1.83** | **9.14** | **1** | **.002** | | **6.25** |  |  |  |  |  |  |
| Psychotic Symptoms | 12.86 | 2.70 | 1 | .100 | | 384782.83 |  |  |  |  |  |  |
| Number of ECT sessions | -0.19 | 3.54 | 1 | .060 | | 0.83 |  |  |  |  |  |  |
| Psychotic Symptoms*ECTs | -0.87 | 2.11 | 1 | .146 | | 0.42 |  |  |  |  |  |  |
| Change Score T0:T1 Item 7 | -0.01 | 2.88 | 1 | .090 | | 0.99 |  |  |  |  |  |  |
|  |  |  |  |  | |  | .30 | 0.70 | 23.73 | 5 | < .001 | 67.7 |
|  |  |  |  |  | |  |  |  |  |  |  |  |
| **Gender** | **1.41** | **6.00** | **1** | **.014** | | **4.11** |  |  |  |  |  |  |
| Psychotic Symptoms | 1.30 | 2.89 | 1 | .089 | | 3.66 |  |  |  |  |  |  |
| **Number of ECT sessions** | **-0.23** | **4.77** | **1** | **.029** | | **0.79** |  |  |  |  |  |  |
| **Change Score T0:T1 Item 8** | **-0.02** | **7.94** | **1** | **.005** | | **0.98** |  |  |  |  |  |  |
|  |  |  |  |  | |  | **.37** | **0.65** | **29.64** | **4** | **< .001** | **71.9** |
|  |  |  |  |  | |  |  |  |  |  |  |  |
| **Gender** | **1.52** | **6.87** | **1** | **.009** | | **4.59** |  |  |  |  |  |  |
| Psychotic Symptoms | 1.45 | 3.36 | 1 | .067 | | 4.25 |  |  |  |  |  |  |
| **Number of ECT sessions** | **-0.20** | **3.90** | **1** | **.048** | | **0.82** |  |  |  |  |  |  |
| Change Score T0:T1 Item 9 | -0.01 | 3.67 | 1 | .055 | | 0.99 |  |  |  |  |  |  |
|  |  |  |  |  | |  | .28 | 0.77 | 21.55 | 4 | < .001 | 72.0 |
|  |  |  |  |  | |  |  |  |  |  |  |  |
| **Gender** | **1.59** | **7.51** | **1** | **.006** | | **4.89** |  |  |  |  |  |  |
| **Psychotic Symptoms** | **1.45** | **3.88** | **1** | **.049** | | **4.26** |  |  |  |  |  |  |
| Number of ECT sessions | 0.01 | 0.00 | 1 | .966 | | 1.01 |  |  |  |  |  |  |
| **ECTs*Change Score T0:T1 Item 10** | **0.01** | **3.95** | **1** | **.047** | | **1.01** |  |  |  |  |  |  |
| **Change Score T0:T1 Item 10** | **-0.07** | **4.81** | **1** | **.028** | | **0.94** |  |  |  |  |  |  |
|  |  |  |  |  | |  | **.29** | **0.62** | **21.29** | **5** | **.001** | **69.2** |
|  |  |  |  |  | |  |  |  |  |  |  |  |
| Gender | 3.08 | 2.33 | 1 | .118 | | 21.73 |  |  |  |  |  |  |
| Psychotic Symptoms | 0.26 | 0.03 | 1 | .857 | | 1.30 |  |  |  |  |  |  |
| Number of ECT sessions | 0.03 | 0.01 | 1 | .909 | | 1.03 |  |  |  |  |  |  |
| **Change Score T0:T2 MADRS Total Score** | **-0.42** | **7.81** | **1** | **.005** | | **0.66** |  |  |  |  |  |  |
|  |  |  |  |  | |  | **.92** | **0.69** | **106.05** | **4** | **< .001** | **93.8** |
|  |  |  |  |  | |  |  |  |  |  |  |  |
| **Gender** | **1.64** | **5.56** | **1** | **.018** | | **5.14** |  |  |  |  |  |  |
| Psychotic Symptoms | 0.48 | 0.34 | 1 | .557 | | 1.62 |  |  |  |  |  |  |
| Number of ECT sessions | -0.08 | 0.43 | 1 | .511 | | 0.93 |  |  |  |  |  |  |
| **Change Score T0:T2 Item 1*** | **-0.07** | **18.94** | **1** | **< .001** | | **0.94** |  |  |  |  |  |  |
|  |  |  |  |  | |  | **.58** | **3.39** | **52.10** | **4** | **< .001** | **81.1** |
|  |  |  |  |  | |  |  |  |  |  |  |  |
| **Gender** | **1.75** | **4.82** | **1** | **.028** | | **5.77** |  |  |  |  |  |  |
| Psychotic Symptoms | 1.06 | 1.30 | 1 | .254 | | 2.89 |  |  |  |  |  |  |
| Number of ECT sessions | -0.22 | 2.90 | 1 | .089 | | 0.81 |  |  |  |  |  |  |
| **Change Score T0:T2 Item 2*** | **-0.10** | **19.98** | **1** | **< .001** | | **0.91** |  |  |  |  |  |  |
|  |  |  |  |  | |  | **.72** | **1.18** | **70.62** | **4** | **< .001** | **84.4** |
|  |  |  |  |  | |  |  |  |  |  |  |  |
| **Gender** | **2.30** | **9.18** | **1** | **.002** | | **9.98** |  |  |  |  |  |  |
| Psychotic Symptoms | 1.51 | 2.69 | 1 | .101 | | 4.51 |  |  |  |  |  |  |
| **Number of ECT sessions** | **-0.24** | **4.91** | **1** | **.027** | | **0.78** |  |  |  |  |  |  |
| **Change Score T0:T2 Item 3*** | **-0.06** | **16.74** | **1** | **< .001** | | **0.94** |  |  |  |  |  |  |
|  |  |  |  |  | |  | **.58** | **1.60** | **51.92** | **4** | **< .001** | **78.1** |
|  |  |  |  |  | |  |  |  |  |  |  |  |
| **Gender** | **1.28** | **4.76** | **1** | **.029** | | **3.58** |  |  |  |  |  |  |
| Psychotic Symptoms | 1.19 | 2.27 | 1 | .132 | | 3.27 |  |  |  |  |  |  |
| Number of ECT sessions | -0.15 | 1.92 | 1 | .166 | | 0.86 |  |  |  |  |  |  |
| **Change Score T0:T2 Item 4*** | **-0.03** | **11.29** | **1** | **.001** | | **0.97** |  |  |  |  |  |  |
|  |  |  |  |  | |  | **.41** | **1.18** | **32.23** | **4** | **< .001** | **75.3** |
|  |  |  |  |  | |  |  |  |  |  |  |  |
| **Gender** | **1.51** | **7.00** | **1** | **.008** | | **4.54** |  |  |  |  |  |  |
| Psychotic Symptoms | 1.37 | 3.28 | 1 | .070 | | 3.91 |  |  |  |  |  |  |
| **Number of ECT sessions** | **-0.31** | **7.96** | **1** | **.005** | | **0.74** |  |  |  |  |  |  |
| **Change Score T0:T2 Item 5** | **-0.01** | **5.15** | **1** | **.023** | | **0.99** |  |  |  |  |  |  |
|  |  |  |  |  | |  | **.30** | **0.83** | **22.84** | **4** | **< .001** | **66.3** |
|  |  |  |  |  | |  |  |  |  |  |  |  |
| **Gender** | **2.99** | **11.44** | **1** | **.001** | | **19.94** |  |  |  |  |  |  |
| **Psychotic Symptoms** | **2.34** | **6.28** | **1** | **.012** | | **10.37** |  |  |  |  |  |  |
| **Number of ECT sessions** | **-0.25** | **4.26** | **1** | **.039** | | **0.78** |  |  |  |  |  |  |
| **Change Score T0:T2 Item 6*** | **-0.05** | **17.85** | **1** | **< .001** | | **0.95** |  |  |  |  |  |  |
|  |  |  |  |  | |  | **.61** | **0.65** | **54.66** | **4** | **< .001** | **76.8** |
|  |  |  |  |  | |  |  |  |  |  |  |  |
| **Gender** | **1.60** | **6.18** | **1** | **.013** | | **4.96** |  |  |  |  |  |  |
| Psychotic Symptoms | 0.76 | 0.83 | 1 | .363 | | 2.13 |  |  |  |  |  |  |
| **Number of ECT sessions** | **-0.73** | **4.26** | **1** | **.039** | | **0.48** |  |  |  |  |  |  |
| **ECTs*Change Score T0:T2 Item 7** | **-0.01** | **2.68** | **1** | **.101** | | **0.99** |  |  |  |  |  |  |
| Change Score T0:T2 Item 7 | 0.06 | 0.92 | 1 | .337 | | 1.07 |  |  |  |  |  |  |
|  |  |  |  |  | |  | .54 | 1.25 | 47.29 | 5 | < .001 | 82.3 |
|  |  |  |  |  | |  |  |  |  |  |  |  |
| Gender | 1.39 | 2.87 | 1 | .090 | | 4.01 |  |  |  |  |  |  |
| Psychotic Symptoms | 0.26 | 0.06 | 1 | .810 | | 1.30 |  |  |  |  |  |  |
| **Number of ECT sessions** | **1.20** | **4.69** | **1** | **.030** | | **3.33** |  |  |  |  |  |  |
| **ECTs*Change Score T0:T2 Item 8** | 0.02 | **6.11** | **1** | **.013** | | **1.02** |  |  |  |  |  |  |
| **Change Score T0:T2 Item 8*** | **-0.36** | **8.28** | **1** | **.004** | | **0.70** |  |  |  |  |  |  |
|  |  |  |  |  | |  | **.72** | **1.08** | **70.19** | **5** | **< .001** | **84.4** |
|  |  |  |  |  | |  |  |  |  |  |  |  |
| **Gender** | **2.03** | **7.75** | **1** | **.005** | | **7.60** |  |  |  |  |  |  |
| Psychotic Symptoms | 0.13 | 0.02 | 1 | .890 | | 1.13 |  |  |  |  |  |  |
| **Number of ECT sessions** | **-0.24** | **4.04** | **1** | **.044** | | **0.78** |  |  |  |  |  |  |
| **Change Score T0:T2 Item 9*** | **-0.05** | **16.79** | **1** | **< .001** | | **0.95** |  |  |  |  |  |  |
|  |  |  |  |  | |  | **.56** | **1.60** | **51.93** | **4** | **< .001** | **82.8** |
|  |  |  |  |  | |  |  |  |  |  |  |  |
| Gender | 4.88 | 2.37 | 1 | .124 | | 131.22 |  |  |  |  |  |  |
| **Psychotic Symptoms** | 1.44 | **3.86** | **1** | **.050** | | **4.22** |  |  |  |  |  |  |
| Number of ECT sessions | 0.24 | 0.33 | 1 | .565 | | 1.27 |  |  |  |  |  |  |
| Gender*ECTs | -0.25 | 1.16 | 1 | .282 | | 0.78 |  |  |  |  |  |  |
| **Change Score T0:T2 Item 10** | **-0.01** | **4.18** | **1** | **.041** | | **0.99** |  |  |  |  |  |  |
|  |  |  |  |  | |  | **.32** | **1.13** | **24.51** | **5** | **< .001** | **71.0** |
|  |  |  |  |  | |  |  |  |  |  |  |  |

*Note.* *R*^2^ = Nagelkerkes *R*^2^. % = Percentage of correct predicition. ECTs = Number of ECTs. Significant predictors and models including significant MADRS predictors are indicated in bold. *Bonferroni-corrected significance. *f* = Cohen’s *f.*

###### Table 7

*Results for the stepwise linear regression analysis, predicting overall symptom reduction*

|  |  | |  |  | Model 2 |  |  | |  | |  |
| --- | --- | --- | --- | --- | --- | --- | --- | --- | --- | --- | --- |
| Variables | ß | *t* | *p* | *F* | *df* | *p* | | *R*^2^ | | *f* | |
| Step 1 |  |  |  |  |  |  | |  | |  | |
| **Gender** | **-0.25** | **-2.59** | **.011** |  |  |  | |  | |  | |
| **Psychotic Symptoms** | **-0.27** | **-2.76** | **.007** |  |  |  | |  | |  | |
| Number of ECT sessions | 0.16 | 1.61 | .111 |  |  |  | |  | |  | |
|  |  |  |  | 4.99 | 3.92 | .003 | | .11 | | 0.35 | |
|  |  |  |  |  |  |  | |  | |  | |
| Step 2 |  |  |  |  |  |  | |  | |  | |
| **Gender** | **-0.24** | **-2.57** | **.012** |  |  |  | |  | |  | |
| **Psychotic Symptoms** | **-0.23** | **-2.51** | **.014** |  |  |  | |  | |  | |
| Number of ECT sessions | 0.11 | 1.22 | .227 |  |  |  | |  | |  | |
| **MADRS Total Score T0*** | **-0.30** | **-3.25** | **.002** |  |  |  | |  | |  | |
|  |  |  |  | **6.77** | **4.91** | **< .001** | | **.20** | | **0.50** | |
|  |  |  |  |  |  |  | |  | |  | |
| **Gender** | **-0.23** | **-2.46** | **.016** |  |  |  | |  | |  | |
| **Psychotic Symptoms** | **-0.22** | **-2.28** | **.025** |  |  |  | |  | |  | |
| Number of ECT sessions | 0.10 | 1.05 | .295 |  |  |  | |  | |  | |
| **MADRS T0 Item 1** | **-0.24** | **-2.41** | **.018** |  |  |  | |  | |  | |
|  |  |  |  | **5.39** | **4.91** | **.001** | | **.16** | | **0.44** | |
|  |  |  |  |  |  |  | |  | |  | |
| **Gender** | **-0.22** | **-2.33** | **.022** |  |  |  | |  | |  | |
| **Psychotic Symptoms** | **-0.23** | **-2.39** | **.019** |  |  |  | |  | |  | |
| Number of ECT sessions | 0.14 | 1.53 | .130 |  |  |  | |  | |  | |
| **MADRS T0 Item 2** | **-.025** | **-2.60** | **.011** |  |  |  | |  | |  | |
|  |  |  |  | **5.66** | **4.91** | **< .001** | | **.16** | | **0.44** | |
|  |  |  |  |  |  |  | |  | |  | |
| **Gender** | **-0.25** | **-2.58** | **.012** |  |  |  | |  | |  | |
| **Psychotic Symptoms** | **-0.27** | **-2.72** | **.008** |  |  |  | |  | |  | |
| Number of ECT sessions | 0.15 | 1.58 | .118 |  |  |  | |  | |  | |
| MADRS T0 Item 3 | -0.02 | -0.25 | .803 |  |  |  | |  | |  | |
|  |  |  |  | 3.72 | 4.91 | .008 | | .10 | | 0.33 | |
|  |  |  |  |  |  |  | |  | |  | |
| **Gender** | **-0.25** | **-2.58** | **.011** |  |  |  | |  | |  | |
| **Psychotic Symptoms** | **-0.26** | **-2.59** | **.011** |  |  |  | |  | |  | |
| Number of ECT sessions | 0.16 | 1.66 | .100 |  |  |  | |  | |  | |
| MADRS T0 Item 4 | -0.06 | -0.55 | .584 |  |  |  | |  | |  | |
|  |  |  |  | 3.79 | 4.91 | .007 | | .11 | | 0.35 | |
|  |  |  |  |  |  |  | |  | |  | |
| **Gender** | **-0.24** | **-2.42** | **.017** |  |  |  | |  | |  | |
| **Psychotic Symptoms** | **-0.28** | **-2.87** | **.005** |  |  |  | |  | |  | |
| Number of ECT sessions | 0.16 | 1.62 | .108 |  |  |  | |  | |  | |
| MADRS T0 Item 5 | -0.14 | -1.43 | .156 |  |  |  | |  | |  | |
|  |  |  |  | 4.29 | 4.91 | .003 | | .12 | | 0.37 | |
|  |  |  |  |  |  |  | |  | |  | |
| **Gender** | **-0.24** | **-2.42** | **.017** |  |  |  | |  | |  | |
| **Psychotic Symptoms** | **-0.26** | **-2.59** | **.011** |  |  |  | |  | |  | |
| Number of ECT sessions | 0.15 | 1.50 | .136 |  |  |  | |  | |  | |
| MADRS T0 Item 6 | -0.09 | -0.860 | .392 |  |  |  | |  | |  | |
|  |  |  |  | 3.91 | 4.91 | .006 | | .11 | | 0.35 | |
|  |  |  |  |  |  |  | |  | |  | |
| **Gender** | **-0.26** | **-2.71** | **.008** |  |  |  | |  | |  | |
| **Psychotic Symptoms** | **-0.26** | **-2.70** | **.008** |  |  |  | |  | |  | |
| Number of ECT sessions | 0.16 | 1.67 | .098 |  |  |  | |  | |  | |
| MADRS T0 Item 7 | -0.13 | -1.39 | .169 |  |  |  | |  | |  | |
|  |  |  |  | 4.26 | 4.91 | .003 | | .12 | | 0.37 | |
|  |  |  |  |  |  |  | |  | |  | |
| **Gender** | **-0.25** | **-2.63** | **.010** |  |  |  | |  | |  | |
| **Psychotic Symptoms** | **-0.28** | **-2.90** | **.005** |  |  |  | |  | |  | |
| Number of ECT sessions | 0.13 | 1.33 | .186 |  |  |  | |  | |  | |
| **MADRS T0 Item 8** | **-0.23** | **-2.36** | **.020** |  |  |  | |  | |  | |
|  |  |  |  | **5.32** | **4.91** | **.001** | | **.15** | | **0.42** | |
|  |  |  |  |  |  |  | |  | |  | |
| **Gender** | **-0.26** | **-2.70** | **.008** |  |  |  | |  | |  | |
| **Psychotic Symptoms** | **-0.24** | **-2.51** | **.014** |  |  |  | |  | |  | |
| Number of ECT sessions | 0.13 | 1.37 | .175 |  |  |  | |  | |  | |
| **MADRS T0 Item 9** | **-0.19** | **-1.95** | **.054** |  |  |  | |  | |  | |
|  |  |  |  | **4.80** | **4.91** | **.001** | | **.14** | | **0.40** | |
|  |  |  |  |  |  |  | |  | |  | |
| **Gender** | **-0.26** | **-2.70** | **.034** |  |  |  | |  | |  | |
| **Psychotic Symptoms** | **-0.30** | **-3.02** | **.003** |  |  |  | |  | |  | |
| Number of ECT sessions | 0.13 | 1.36 | .176 |  |  |  | |  | |  | |
| MADRS T0 Item 10 | -0.15 | -1.50 | .137 |  |  |  | |  | |  | |
|  |  |  |  | 4.35 | 4.91 | .003 | | .12 | | 0.37 | |
|  |  |  |  |  |  |  | |  | |  | |
| Gender | -0.11 | -1.29 | .202 |  |  |  | |  | |  | |
| Psychotic Symptoms | -0.14 | -1.67 | .098 |  |  |  | |  | |  | |
| Number of ECT sessions | 0.07 | .88 | .383 |  |  |  | |  | |  | |
| **Change Score T0:T1 MADRS Total Score*** | **0.54** | **6.18** | **< .001** |  |  |  | |  | |  | |
|  |  |  |  | **14.80** | **4.91** | **< .001** | | **.37** | | **0.77** | |
|  |  |  |  |  |  |  | |  | |  | |
| Gender | -0.19 | -2.14 | .035 |  |  |  | |  | |  | |
| Psychotic Symptoms | -0.17 | -1.88 | .064 |  |  |  | |  | |  | |
| Number of ECT sessions | 0.06 | .64 | .521 |  |  |  | |  | |  | |
| **Change Score T0:T1 Item 1*** | **0.43** | **4.63** | **< .001** |  |  |  | |  | |  | |
|  |  |  |  | **9.65** | **4.90** | **< .001** | | **.27** | | **0.61** | |
|  |  |  |  |  |  |  | |  | |  | |
| Gender | -0.10 | -1.10 | .273 |  |  |  | |  | |  | |
| Psychotic Symptoms | -0.14 | -1.62 | .108 |  |  |  | |  | |  | |
| Number of ECT sessions | 0.08 | .95 | .343 |  |  |  | |  | |  | |
| **Change Score T0:T1 Item 2*** | **0.54** | **6.17** | **< .001** |  |  |  | |  | |  | |
|  |  |  |  | **14.77** | **4.91** | **< .001** | | **.37** | | **0.77** | |
|  |  |  |  |  |  |  | |  | |  | |
| **Gender** | **-0.33** | **-3.13** | **.002** |  |  |  | |  | |  | |
| **Psychotic Symptoms** | **-0.26** | **-2.81** | **.006** |  |  |  | |  | |  | |
| Number of ECT sessions | 0.16 | 1.75 | .083 |  |  |  | |  | |  | |
| **Gender*Change Score T0:T1 Item 3** | **-0.66** | **-2.20** | **.030** |  |  |  | |  | |  | |
| **Change Score T0:T1 Item 3*** | **0.87** | **3.00** | **.003** |  |  |  | |  | |  | |
|  |  |  |  | **5.98** | **5.90** | **< .001** | | **.21** | | **0.52** | |
|  |  |  |  |  |  |  | |  | |  | |
| **Gender** | **-0.25** | **-2.52** | **.013** |  |  |  | |  | |  | |
| **Psychotic Symptoms** | **-0.27** | **-2.76** | **.007** |  |  |  | |  | |  | |
| Number of ECT sessions | 0.11 | 1.10 | .275 |  |  |  | |  | |  | |
| **Change Score T0:T1 Item 4** | **0.22** | **2.20** | **.031** |  |  |  | |  | |  | |
|  |  |  |  | **5.26** | **4.84** | **< .001** | | **.16** | | **0.44** | |
|  |  |  |  |  |  |  | |  | |  | |
| **Gender** | **-0.25** | **-2.48** | **.015** |  |  |  | |  | |  | |
| **Psychotic Symptoms** | **-0.23** | **-2.32** | **.022** |  |  |  | |  | |  | |
| Number of ECT sessions | 0.16 | 1.59 | .116 |  |  |  | |  | |  | |
| Change Score T0:T1 Item 5 | 0.12 | 1.17 | .245 |  |  |  | |  | |  | |
|  |  |  |  | 3.59 | 4.88 | .009 | | .10 | | 0.33 | |
|  |  |  |  |  |  |  | |  | |  | |
| **Gender** | **-0.22** | **-2.26** | **.026** |  |  |  | |  | |  | |
| **Psychotic Symptoms** | **-0.24** | **-2.49** | **.015** |  |  |  | |  | |  | |
| Number of ECT sessions | 0.15 | 1.63 | .106 |  |  |  | |  | |  | |
| **Change Score T0:T1 Item 6*** | **0.28** | **2.97** | **.004** |  |  |  | |  | |  | |
|  |  |  |  | **6.56** | **4.90** | **< .001** | | **.19** | | **0.48** | |
|  |  |  |  |  |  |  | |  | |  | |
| **Gender** | **-0.24** | **-2.54** | **.013** |  |  |  | |  | |  | |
| **Psychotic Symptoms** | **-0.22** | **-2.30** | **.024** |  |  |  | |  | |  | |
| Number of ECT sessions | 0.16 | 1.67 | .099 |  |  |  | |  | |  | |
| **Change Score T0:T1 Item 7** | **0.23** | **2.34** | **.021** |  |  |  | |  | |  | |
|  |  |  |  | **5.29** | **4.91** | **.001** | | **.15** | | **0.42** | |
|  |  |  |  |  |  |  | |  | |  | |
| **Gender** | **-0.19** | **-2.23** | **.029** |  |  |  | |  | |  | |
| **Psychotic Symptoms** | **-0.23** | **-2.70** | **.008** |  |  |  | |  | |  | |
| Number of ECT sessions | 0.03 | 0.35 | .725 |  |  |  | |  | |  | |
| **ECTs*Change Score T0:T1 Item 8*** | **-1.11** | **-3.35** | **.001** |  |  |  | |  | |  | |
| **Change Score T0:T1 Item 8*** | **1.44** | **4.32** | **< .001** |  |  |  | |  | |  | |
|  |  |  |  | **9.63** | **5.90** | **< .001** | | **.31** | | **0.67** | |
|  |  |  |  |  |  |  | |  | |  | |
| **Gender** | **-0.19** | **-1.95** | **.054** |  |  |  | |  | |  | |
| **Psychotic Symptoms** | **-0.22** | **-2.22** | **.029** |  |  |  | |  | |  | |
| Number of ECT sessions | 0.13 | 1.34 | .185 |  |  |  | |  | |  | |
| **Change Score T0:T1 Item 9** | **0.28** | **2.89** | **.005** |  |  |  | |  | |  | |
|  |  |  |  | **5.48** | **4.88** | **.001** | | **.16** | | **0.44** | |
|  |  |  |  |  |  |  | |  | |  | |
| **Gender** | **-0.23** | **-2.56** | **.012** |  |  |  | |  | |  | |
| **Psychotic Symptoms** | **-0.26** | **-2.81** | **.006** |  |  |  | |  | |  | |
| Number of ECT sessions | -0.17 | -1.36 | .178 |  |  |  | |  | |  | |
| **ECTs*Change Score T0:T1 Item 10*** | **-1.61** | **-3.17** | **.002** |  |  |  | |  | |  | |
| **Change Score T0:T1 Item 10*** | **1.87** | **3.67** | **< .001** |  |  |  | |  | |  | |
|  |  |  |  | **7.22** | **5.85** | **< .001** | | **.26** | | **0.59** | |
|  |  |  |  |  |  |  | |  | |  | |
| Gender | 0.09 | 0.77 | .443 |  |  |  | |  | |  | |
| Psychotic Symptoms | -0.07 | -1.14 | .258 |  |  |  | |  | |  | |
| Number of ECT sessions | -0.06 | -0.95 | .344 |  |  |  | |  | |  | |
| **Gender*Change Score T0:T2 Item 1** | **0.49** | **2.02** | **.046** |  |  |  | |  | |  | |
| Change Score T0:T2 Item 1 | 0.37 | 1.75 | .084 |  |  |  | |  | |  | |
|  |  |  |  | 34.30 | 5.89 | < .001 | | .64 | | 1.42 | |
|  |  |  |  |  |  |  | |  | |  | |
| Gender | -0.04 | -0.68 | .497 |  |  |  | |  | |  | |
| **Psychotic Symptoms** | **-0.11** | **-2.06** | **.042** |  |  |  | |  | |  | |
| **Number of ECT sessions** | **-0.27** | **-2.65** | **.009** |  |  |  | |  | |  | |
| **ECTs*Change Score T0:T2 Item 2*** | **-1.04** | **-3.44** | **.001** |  |  |  | |  | |  | |
| **Change Score T0:T2 Item 2*** | **1.84** | **6.08** | **< .001** |  |  |  | |  | |  | |
|  |  |  |  | **55.81** | **5.90** | **< .001** | | **.74** | | **1.69** | |
|  |  |  |  |  |  |  | |  | |  | |
| Gender | -0.02 | -0.19 | .849 |  |  |  | |  | |  | |
| **Psychotic Symptoms** | **-0.19** | **-2.51** | **.014** |  |  |  | |  | |  | |
| Number of ECT sessions | 0.12 | 1.53 | .129 |  |  |  | |  | |  | |
| Gender*Change Score T0:T2 Item 3 | 0.49 | 1.87 | .064 |  |  |  | |  | |  | |
| Change Score T0:T2 Item 3 | 0.17 | 0.70 | .485 |  |  |  | |  | |  | |
|  |  |  |  | 17.61 | 5.90 | < .001 | | .47 | | 0.94 | |
|  |  |  |  |  |  |  | |  | |  | |
| **Gender** | **-0.20** | **-2.16** | **.033** |  |  |  | |  | |  | |
| **Psychotic Symptoms** | **-0.23** | **-2.45** | **.016** |  |  |  | |  | |  | |
| Number of ECT sessions | **0.08** | 0.82 | .412 |  |  |  | |  | |  | |
| **Change Score T0:T2 Item 4*** | **0.34** | **3.54** | **.001** |  |  |  | |  | |  | |
|  |  |  |  | **7.23** | **4.88** | **< .001** | | **.21** | | **0.52** | |
|  |  |  |  |  |  |  | |  | |  | |
| Gender | -0.18 | -1.94 | .055 |  |  |  | |  | |  | |
| **Psychotic Symptoms** | **-0.22** | **-2.43** | **.017** |  |  |  | |  | |  | |
| **Number of ECT sessions** | **0.24** | **2.67** | **.009** |  |  |  | |  | |  | |
| **Change Score T0:T2 Item 5*** | **0.44** | **4.78** | **< .001** |  |  |  | |  | |  | |
|  |  |  |  | **9.90** | **4.87** | **< .001** | | **.28** | | **0.62** | |
|  |  |  |  |  |  |  | |  | |  | |
| Gender | -0.08 | -0.86 | .391 |  |  |  | |  | |  | |
| **Psychotic Symptoms** | **-0.25** | **-3.26** | **.002** |  |  |  | |  | |  | |
| Number of ECT sessions | -0.04 | -0.42 | .675 |  |  |  | |  | |  | |
| **Gender*Change Score T0:T2 Item 6** | **0.61** | **2.52** | **.013** |  |  |  | |  | |  | |
| **ECTs*Change Score T0:T2 Item 6** | **-1.21** | **-2.61** | **.011** |  |  |  | |  | |  | |
| **Change Score T0:T2 Item 6** | **1.18** | **2.20** | **.031** |  |  |  | |  | |  | |
|  |  |  |  | **15.37** | **6.88** | **< .001** | | **.48** | | **0.96** | |
|  |  |  |  |  |  |  | |  | |  | |
| Gender | -0.13 | -1.65 | .103 |  |  |  | |  | |  | |
| Psychotic Symptoms | -0.15 | -1.92 | .058 |  |  |  | |  | |  | |
| Number of ECT sessions | 0.07 | 0.94 | .348 |  |  |  | |  | |  | |
| **Change Score T0:T2 Item 7*** | **0.61** | **7.72** | **< .001** |  |  |  | |  | |  | |
|  |  |  |  | **21.02** | **4.91** | **< .001** | | **.46** | | **0.92** | |
|  |  |  |  |  |  |  | |  | |  | |
| **Gender** | **-0.16** | **-2.42** | **.018** |  |  |  | |  | |  | |
| Psychotic Symptoms | -0.12 | -1.81 | .073 |  |  |  | |  | |  | |
| **Number of ECT sessions** | **0.15** | **2.39** | **.019** |  |  |  | |  | |  | |
| **Change Score T0:T2 Item 8*** | **0.72** | **10.91** | **< .001** |  |  |  | |  | |  | |
|  |  |  |  | **38.29** | **4.91** | **< .001** | | **.61** | | **1.25** | |
|  |  |  |  |  |  |  | |  | |  | |
| **Gender** | **-0.19** | **-2.72** | **.008** |  |  |  | |  | |  | |
| Psychotic Symptoms | -0.05 | -0.74 | .463 |  |  |  | |  | |  | |
| **Number of ECT sessions** | **0.21** | **2.95** | **.004** |  |  |  | |  | |  | |
| **Change Score T0:T2 Item 9*** | **0.71** | **9.74** | **< .001** |  |  |  | |  | |  | |
|  |  |  |  | **30.14** | **4.88** | **< .001** | | **.56** | | **1.13** | |
|  |  |  |  |  |  |  | |  | |  | |
| **Gender** | **-0.24** | **-2.55** | **.013** |  |  |  | |  | |  | |
| **Psychotic Symptoms** | **-0.25** | **-2.77** | **.007** |  |  |  | |  | |  | |
| Number of ECT sessions | **0.11** | 1.11 | .271 |  |  |  | |  | |  | |
| **Change Score T0:T2 Item 10*** | **0.31** | **3.18** | **.002** |  |  |  | |  | |  | |
|  |  |  |  | **7.98** | **4.88** | **< .001** | | **.23** | | **0.55** | |
|  |  |  |  |  |  |  | |  | |  | |

*Note.* *R*^2^ = adjusted *R*^2,^ . ECTs = Number of ECTs. ß = standardized ß-value. Significant predictors and models including significant MADRS predictors are indicated in bold. *Bonferroni-corrected significance. *f* = Cohen’s *f.*
